# Learning zero-cost portfolio selection with pattern matching

**DOI:** 10.1371/journal.pone.0202788

**Published:** 2018-09-25

**Authors:** Fayyaaz Loonat, Tim Gebbie

**Affiliations:** 1 School of Computer Science and Applied Mathematics, University of the Witwatersrand, Johannesburg, WITS 2050, South Africa; 2 Department of Statistical Sciences, University of Cape Town, Cape Town, Rondebosch 7701, South Africa; UCLA, UNITED STATES

## Abstract

We replicate and extend the adversarial expert based learning approach of Györfi *et al* to the situation of zero-cost portfolio selection implemented with a quadratic approximation derived from the mutual fund separation theorems. The algorithm is applied to daily sampled sequential Open-High-Low-Close data and sequential intraday 5-minute bar-data from the Johannesburg Stock Exchange (JSE). Statistical tests of the algorithms are considered. The algorithms are directly compared to standard NYSE test cases from prior literature. The learning algorithm is used to select parameters for experts generated by pattern matching past dynamics using a simple nearest-neighbour search algorithm. It is shown that there is a speed advantage associated with using an analytic solution of the mutual fund separation theorems. We argue that the strategies are on the boundary of profitability when considered in the context of their application to intraday quantitative trading but demonstrate that patterns in financial time-series on the JSE could be systematically exploited in collective and that they are persistent in the data investigated. We do not suggest that the strategies can be profitably implemented but argue that these types of patterns may exists for either structural of implementation cost reasons.

## Introduction

Sequential investment strategies aim to facilitate portfolio control decisions by collecting information from past behaviour and states of the market and using this information to deploy capital across a selection of assets in a manner the can generate consistent wealth maximization over the long-term [1–4]. These methods may be useful in probing the structure of financial markets as they have the ability to learn structure from financial time-series data using real-time streaming time-series data.

The intention of the paper is not to find a profitable trading strategy for quantitative trading but to add additional examples to the data-intensive literature of the application of a simple, transparent and easily recoverable strategy on the boundary of profitability while extending the existing application of pattern-matching to the case of self-funding strategies that can be directly compared to a risk-free asset and thus used to probe the boundaries of arbitrage. The intention is to show that predictable strategies exists even if they reside on the boundary of profitability. We argue that the behaviour of these strategies can be well understood using surrogate data in combination with real world data and that the algorithm behaviour cannot be attributed to randomness.

We make no specific assumptions relating to the nature of price processes for the sake of the algorithms, however, the approach is broadly based on prior mathematical analysis that use assumptions of stationarity and ergodicity of the price increments in order to allow the study of asymptotic growth rates. In particular to ensure that such growth rates have well-defined maxima when full knowledge of the distribution and its process have been achieved [1, 2, 4, 5].

We do not make any explicit assumptions about the behaviour of the experts because we do not implement explicit trading rules. The only expert advice provided is that of testing of the persistence of predictability from similar historical patterns fitted to the past [4] and preferential selected for using an online multiplicative weighting algorithm [2]. Here the success (or failure) of the predictions is measured by the accumulated wealth of the online algorithm.

There is a rich and diverse literature on the online portfolio decision making in the context of sequential trading and investment strategies [6]. Although these methods are directly related to the learning algorithm used in the approach used here [4]. The pattern matching algorithm itself is not an online algorithm as the entire history of the data is searched by the pattern matching algorithm at each time-step.

The motivation for the selection of the pattern matching method in conjunction with the online learning algorithm was based on the requirement of having a simple testable and provable learning algorithm, that was online, that could be applied to a simple pattern matching algorithm that could be used to generate equity curves—profit and loss curves through time. This is the simplest brute-force pattern matching algorithm that we were able to find that could be easily integrated with the online learning algorithm. This type of algorithm implementation is well suited as a base-line strategy for explicit experts that can be used to directly probe market structure and compared with experts when the trading rules are known.

For these reasons we further investigated the well established idea that by using pattern matching algorithms combined with learning algorithms based on the purpose of wealth maximisation irrespective or risk [1, 7] we can:

1. Beat a cash portfolio in the context of a self-funding strategy, a zero-cost portfolio strategy, and that
2. We can beat the best stock in the market [8].

The latter has been shown to be the case in prior literature, by investigating daily sampled stock data from the NYSE for long-only (fully invested) portfolio strategies [1, 2, 8–10]. The prior has been considered in literature in the context of long-short investing with the inclusion of a margin account [11] as well as naked short selling [5]. We explicitly consider leverage one self-funding portfolios rather than margined trading, naked long-short investing or the inclusion of a cash-asset against which the trader can borrow and do so for an implementation of pattern matching based on an analytic approximation.

We also consider both of these cases on a novel data-set: zero-cost, and fully invested strategies, in the context of the South African stock market, the Johannesburg Stock Exchange (JSE), and do so for both daily sampled data and intraday data.

We are not searching for statistically preserved properties of time-series data in the sense of time-series models but are looking for evidence of statistical repeating structures in time-series data without *a-priori* ability to know the form that the structure will take [12, 13]. The patterns are always unknown, changing and dynamic and are approximated from the collective past histories of the system components.

The appearance of patterns and organisation is a fundamental property of complex adaptive systems [14]. Looking directly for pockets of predictability in complex dynamical systems [15] as an approximation to modelling complex adaptive systems [14] is notoriously difficult given the intricacies of noise and nonlinearity [16, 17]. It is this which motivated our framework to look for pockets of predictability, if they exist, via pattern searching in order to increase our experts wealth irrespective of risk.

By extracting positive growth rates in the excess of the performance of the best stock by using unleveraged combinations of underlying stocks over long periods of time we build the case that there are patterns, some sort of structures, that almost repeat though time in a manner that their occurrence can be treated as exploitable information. This has been shown to be the case for long-only portfolio’s [1, 2, 8–10]. We show this for self-funding strategies; zero-cost portfolio’s. This allows us to relate the strategies to various estimates of transaction costs while providing a framework which can both be easily related to prior literature through replication, and be proved using surrogate data.

We do not address the question of whether it is risk that the investor is being compensated for or whether the strategies we are isolating are statistical arbitrages; here in the sense that the strategies long-term volatility tends to zero in conjunction with an always positive probability of positive performance at zero initial cost [18].

In the section An Online-Learning Algorithm for Portfolio Selection, we present the expert learning algorithm as an extension of prior work [1, 2, 5, 7] and [4, 10]. The contributions here are: (i) the algorithm is explicitly re-written in online form in order to make near-real-time applications tractable, (ii) the algorithms are modified for application to the zero-cost portfolio selection problem using the mutual fund separation theorems [19, 20], (iii) the algorithms are explicitly tested, using synthetic data, real daily data both from the NYSE and JSE, and for JSE intraday 5-minute bar-data.

The Expert Generating Algorithms section describes the approach we have adopted for the generation of experts in zero-cost portfolio strategies. The algorithm parameters are not tuned prior to use but are left to the online-learning algorithm to select.

In the section Expert Generating Algorithms from Patterns, we consider strategies that target predictable patterns using a simple modified version of the nearest-neighbour pattern-matching strategy developed by [4].

As in the case of the learning algorithm, the expert-generation algorithms have been modified in principle: (i) to support offline and online algorithm use, (ii) they are explicitly framed for use with zero-cost portfolio selection problems, and (iii) portfolio optimizations have been replaced with analytic quadratic approximations in order to improve execution times.

In order to have true online pattern matching the algorithms would have to be replaced with either look-up-tables built off-line or a hybrid method that combines offline building of the history of the experts performance and then an almost online method that updates that cached history of experts performance across parameters as the data arrives sequentially in real-time.

The Data Description section provides an overview of the data used in the various numerical experiments.

The data is sequential and uniformly sampled and takes on the form of open-high-low-close (OHLC) data, this is described in the OHLC Data section. The use of open, high, low and close data combinations for the daily data testing can be carried over for intraday studies, and the use of close prices is a special case.

The synthetic data is described in the Synthetic Data section along with the algorithm testing strategy. Briefly, a simple Kolmogorov-Smirnov test is adopted to assess algorithm behaviour across 4 test cases:

1. SDC1: log-normal random data with zero-means, where no learning should be possible,
2. SDC2: log-normal random data where all assets have the same positive mean and as such basic learning is not possible for zero-cost portfolios (portfolios that have long and short positions that sum to zero),
3. SDC3: log-normal random data with varying positives means, and
4. SDC4: where we have log-normal data with both positive and negative means with the same fixed variance.

The synthetic data is used to understand and prove the behaviour of the zero-cost portfolio strategy (which we will call active portfolio’s) and the fully-invested portfolio strategy (which we will call absolute portfolio’s).

The four real-world data sets are described in the Real Data section:

1. The standard daily sampled test-data set for the NYSE [1, 2, 8–10],
2. A more extensive, merged, daily sampled test-data set for the NYSE [21],
3. A daily sampled test-data set for the JSE, and
4. An intraday test-data set for the JSE.

A general overview of the implementation of the numerical experiments is addressed in the Implementation section. The Impact of Market Frictions section discusses market frictions in terms of direct and indirect costs [22]. In the Conclusion we suggest that our work supports the general argument that fairly naive data-informed computational learning experts with the appropriate access to the systems can at least cover the cost of daily sampled trading on the JSE without special insights.

## An online-learning algorithm for portfolio selection

The application is for a set of stocks ordered in time where each expert will consider different combinations of stocks for each time-period based on features and strategy parameters. These different experts compete in an adversarial manner in competition for capital allocations [2–5, 7]. Here experts with poor performance will have incremental capital allocations reduced and experts with robust performance will have incremental increases in capital allocation. Better performing experts will over time have their relative contribution to the aggregate portfolio increased so that their decisions are preferentially selected for trade at the onset of each trading or investment period based on information available at the end of the prior trading period.

The online learning algorithm takes as inputs: a set of experts controls, and performances. These are enumerated over features (here price-relatives) and free-parameters of the temporally ordered objects (here stocks).

The key feature used will be price relatives which are defined for the *m*-th object as:

$$\begin{array}{c} x_{m,t}=\frac{p_{m,t}}{p_{m,t-1}} \end{array}$$ (1)

In vector notation we will write this equivalently as ***x***_*t*_ where the *m*-th component is *x*_*m*,*t*_.

The controls that represent the experts’ are the portfolio weights by which each expert’s decision will contribute to the final aggregate decision at a particular time.

Expert performance is represented by factor (expert) mimicking portfolios that are formed from the portfolio controls at each time period. The controls are estimated and implemented at the beginning of each period. The relative changes in asset performance will then modify the relative weights of the asset over the investment period and the performance of a given expert is then determined at the end of the investment period.

This is determined both by the controls, and selecting for the collection of objects the expert is holding, their weights, and the performance of those objects as determined by price relatives.

Experts do not have to hold the same number of objects. Experts can hold all or small groups of objects, they can short-sell objects and hold long positions in objects. Short-selling is when an asset is borrowed for a small fee, and the capital raised from the sale can then be used for other trading or investment activities, for example, the raised capital can be used to buy another asset by taking a long-position. The combination of long and short positions can be cash-neutral where the total value of the initial portfolio is zero. Such a portfolio is called a zero-cost or cash-neutral portfolio. The collection of objects a particular expert holds will be called the expert’s object cluster.

The parameters that denote experts are typically a parameter that is an index of the cluster of objects an expert has decided to use, and the algorithm specific parameters; typically a data window parameter *k* determining how much past data to include, and a parameter more specific to a given algorithm if it is required, such as a partition parameter *ℓ*, and a forecast horizon dependent parameter, *τ*.

Any four useful parameters can be used in the learning algorithm that was implemented in this paper. The number of experts is then a function of these four free-parameters. The learning algorithm will then carry out the weighted averaging process based on expert past performance over the experts enumerated by these four parameters.

The parameters are denoted *τ*, *w*, *k* and *ℓ* respectively. We reserved parameters *k* and *ℓ* for algorithm specific parameters—this is done in order to try to align with their usage in the prior literature [10]. There are at most *W* values of *w*, *K* values of *k*, *L* values of *ℓ* and *τ*_*n*_ values for the horizon parameter *τ*.

The default value of the horizon parameter is 1: *τ* = 1. For simplicity and computational speed the results presented in this paper have used the default value. It is anecdotally noted that there is an advantage in learning for the horizon parameter but this does not change the basic point made in this paper. The choice of these parameters will determine the number of experts in the system. The number of experts is denoted by *n* where the total number of experts will then be *N* = *τ*_*n*_*WKL*.

The *n*-th expert is represented by a tuple containing the controls at a given time and its performance (*H*_*nm*,*t*_, *S*_*n*,*t*_). This tuple will usually be represented in vector notation as (***H***_*n*,*t*_, *S*_*n*,*t*_) where the object index *m* is suppressed.

For discrete values of sequential time running from *t* = 1 until some maximal time *T* the expert controls ***H*** are then collection of *T* time-ordered (*N*, *M*)-dimensional matrices that are represented as multi-dimensional double precision matrices in the software.

The value of the *n*-th experts controls for the *m*-th object at time *t* is *H*_*nm*,*t*_ for discrete values of time. The performance of the experts is represented as a (*N*, *T*)-dimensional matrix where the *n*-th expert has its performance over the *t*-th time interval as *S*_*n*,*t*_.

There are at most *M* objects. So *m* can take on values on the integer interval [1, *M*] that would enumerate the objects. The number of objects remain static for a given expert even though they may be able to achieve zero positions in a particular expert.

From the perspective of the learning algorithm the mechanism of expert generation is not important, it is required that all *N* experts are correctly enumerated at each time increment. At the beginning of each time increment the controls determined at the end of the previous time increment are implemented and then held to the end of the time period at which time the expert performance is determined and the expert controls are then adjusted using the learning algorithm.

The learning algorithm updates the expert mixture control *q*_*n*,*t*_ which is a measure of how much a given expert will contribute to the aggregate portfolio. The *q* variables control the relative mixture of experts through time as they compete based on their past performance. The mixture controls cannot in general be thought of as probabilities, which makes their use and notation different to some of the prior literature [10].

### Online-learning algorithm

The learning algorithm (see S1 Appendix) is inspired by the universal portfolio approach developed by [2, 5] and refined by [4]. The learning expert can be thought of as a multi-manager, using asset management language, where the multi-manager is selecting and aggregating underlying strategies from a collection of portfolios ***H***_*n*,*t*_ and then aggregating using some selection method to a single portfolio ***b***_*t*_ that is implemented at each investment or trading period *t*.

The basic learning algorithm was incrementally implemented online, but offline it can be easily parallelized across experts. The learning algorithm has five key steps:

1. **Update the portfolio wealth**: The portfolio controls *b*_*m*,*t*_ for the *m*-th asset are used to update the portfolio returns for the *t*-th period
  $$\Delta S_{t}=[\sum_{m}b_{m,t}\left(x_{m,t}-1\right)]+1$$ (2)
  $$S_{t}=S_{t-1}\Delta S_{t}.$$ (3)
  Here the price relatives for the *t*-th period and *m*-th asset, *x*_*m*,*t*_, are combined with the portfolio controls for the period just ending to compute the realised portfolio returns for this period, period *t*. The portfolio controls were computed at the end of the prior period and implemented at the beginning of the current period. The relative amounts of each object in the portfolio will have changed by the relative price changes assuming no cash-flows into or out of the portfolio during this investment period.
2. **Update expert wealth**: The expert controls *H*_*nm*,*t*_ were determined at the end of time-period *t* − 1 for time period *t* by some expert generating algorithm for *N* experts and *M* objects about which the experts make expert capital allocation decisions. At the end of the *t*-th time period the performance of each expert, *S*_*n*,*t*_, can be computed from the change in the price relatives *x*_*m*,*t*_ for the each of the *M* objects in the investment universe considered using the prices at the start, *p*_*m*, *t*−1_, and the end of the *t*-th time increment, *p*_*m*,*t*_, using the expert controls.
  $$\Delta S_{n,t}=[\sum_{m}H_{nm,t}\left(x_{m,t}-1\right)]+1.$$ (4)
  $$S_{n,t}=S_{n,t-1}\Delta S_{n,t}.$$ (5)
3. **Update expert mixtures**: We considered three different expert mixture update rules: 1.) the universally consistent choice, and 2.) an exponential gradient choice [23] and 3.) an exponentially weighted moving average. We generically refer to these online updates as rule *g*. In practice one would select one of the three update rules once for the duration of the offline training, if one seeks to initialise the algorithm prior to deployment, or for use online during the system implementation in real-time. For the numerical experiments presented here we adopted the universal consistent approach inspired by [5] and [4] as this demonstrates the principle. We can define the mixture of controls as the accumulated expert wealth is used as the update feature for the next unrealised increment with some normalisation, as such, the expert mixture control for the *n*-th expert for the next time increment, *t* + 1, is proportional to the measure of wealth:
  $$q_{n,t+1}\propto S_{n,t}.$$ (6)
  the alternative choices can include the Exponential Gradient (EG) based learning approach of [23]:
  $$q_{n,t+1}=q_{n,t}e^{\left(\frac{\eta S_{n,t}}{\sum_{n}\;q_{n,t}S_{n,t}}\right)}$$ (7)
  or an Exponential Weighted Moving Average (EWMA) based learning strategy:
  $$q_{n,t+1}=\lambda q_{n,t}+\left(1-\lambda \right)(\frac{q_{n,t}S_{n,t}}{\sum_{n}\;q_{n,t}S_{n,t}})$$ (8)
  We adopt the simplest update rule for the mixture of controls, it should be noted that there can be practical advantages to using more adaptive methods such as EG and EWMA learning where the learning rates can be used as additional parameters to be learnt using a thick modelling framework [9].
4. **Re-normalise expert mixtures**: If the expert mixture is to be considered a positive probability then we require that ∑_*n*_ *q*_*n*_ = 1 and that all *q*_*n*_ ≥ 0. This is the case of fully-invested experts where no shorting is allowed. We will call these types of experts *absolute* experts:
  $$\begin{array}{c} q_{n,t+1}=\frac{q_{n,t+1}}{\sum_{n}q_{n,t+1}}. \end{array}$$ (9)
  For experts that we will consider *active* the leverage is set to unity for zero-cost portfolios: (1.) ∑_*n*_ *q*_*n*_ = 0 and (2.) *ν* = ∑_*n*_ |*q*_*n*_| = 1. Here the mixture controls allow for shorting of one expert against another and the portfolio becomes self-funding. The mixture controls can no-longer be thought of as positive probabilities.
  $$\begin{array}{c} q_{n,t+1}=\frac{q_{n,t+1}-\frac{1}{N}\sum_{n}q_{n,t+1}}{\sum_{n}\left|q_{n,t+1}-\frac{1}{N}\sum_{n}q_{n,t+1}\right|} \end{array}$$ (10)
  The leverage is normalised in order to ensure consistency between the learning algorithms and expert generating algorithms.
5. **Update portfolio controls**: The portfolio controls *b*_*m*,*t*_ are updated at the end of time period *t* for time period *t* + 1 using the expert mixture controls *q*_*n*,*t*+1_ from the updated learning algorithm and the expert controls *H*_*nm*,*t*+1_ from the expert generating algorithms using information from time period *t* and averaged over all *n* experts.
  $$\begin{array}{c} b_{m,t+1}=\sum_{n}q_{n,t+1}H_{nm,t+1}. \end{array}$$ (11)

The strategy is to implement the portfolio controls, wait until the end of the increment, measure the features, update the experts and then re-apply the learning algorithm to compute the expert mixtures and portfolio controls for the next time increment.

## Expert generating algorithms

The purpose of the expert generating algorithms are to sequentially generate the expert controls *H*_*nm*,*t*_ for the *n*-th expert for the *m*-th object for implementation at the start of the *t*-th time period. These will be denoted in vector notation as ***H***_*n*,*t*_.

We initially considered three different expert-generating algorithms over which the thick modelling was carried out in order to learn the various algorithms’ free-parameters: 1.) a pattern-matching algorithm [10], 2.) a contrarian mean-variance portfolio algorithm we called anti-BCRP (as it trades against the Best Constant Rebalanced Portfolio for a given k-tuple of data) which can be used to learn for mean-reversion by directly using k past realisations of performance of each object, for a given partition, by finding the mean-variance wealth minimizing portfolio (in order to be contrarian), either fully-invested or zero-cost, and using the resulting portfolio weights for the experts with the specific window and partition parameters, ***H***_*n*,*t*+1_ = ***H***_*n*,*t*+1_(*γ*, −***μ***(***x***_*n*,*t*_), Σ(***x***_*n*,*t*_)), comparing with Eqs (27) and (28), and 3.) the ANTICOR algorithm [8]. The various free-parameters of these algorithms, such as the window sizes k and partitions *ℓ* were then used to enumerate the experts that would compete for capital allocations in the learning algorithm.

We adopted the pattern-matching approach [10] for the numerical experiments in this paper as we found a performance advantage in looking for more general patterns rather than merely targeting mean-reversion effects, and more importantly, the pattern-matching algorithms are more generic as they do not require any *a-priori* choices for the structures that are learnt for. This was considered to be more faithful to the intent of the paper—where we seek to show that unspecified patterns can be learnt for in a manner that can both beat the best single stock in a universe of stocks and can beat a cash portfolio in a self-funding strategy.

### Comments on notation

The feature realisations at time *t* for the *m*-th object, *x*_*m*,*t*_, are also denoted in vector notation as ***x***_*t*_. The expert controls and the feature time-series are the key inputs in the online-learning algorithm to determine the expert mixtures *q*_*n*,*t*_ through time. The online learning algorithm is path-dependent and as such both a function of the history of expert controls as well as the feature time-series history.

Following prior work we denote random feature variables as ***X*** and their realisations as ***x*** [3, 4, 10] where for some vector valued stationary and ergodic process $\{X_{t}{\}}_{-\infty }^{+\infty }$ with realisations denoted as ***x***_1_, ***x***_2_, …, ***x***_*t*_ and their corresponding random variables as ***X***_1_, ***X***_2_, …, ***X***_*t*_. However, we will refine the notation further in order to more effectively enumerate the experts for our specific implementation.

The strategies are based on constructing a *k*-tuple of the selected feature for *m*-objects. We will denote the expert-tuple by ***x***_*kℓw*,*t*_ and the *k*-tuple as $x_{t}^{t-k}$. The *k*-tuple is a slice of data of length *k* from the current time *t*, of width *m* enumerating all the objects. We will modify the *k*-tuple notation to $\{x_{t}^{t-k}{\}}_{s(n),\ell }$ to denote a *k*-tuple taken from an *ℓ*-partition of the data for a given cluster of objects *w* = *s*(*n*). Here *s* is the cluster index of the *n*-th expert. We are suppressing the *m* index and using vector notation to write the *k*-tuple as ***x***. The expert-tuple will be unique to the *n*-th expert where *n* is the unique expert index enumerating a particular combination of *k*, *ℓ* and *w*.

A *k*-tuple is used to determine expert controls ***H***_*n*,*t*_. The initial features used are historical prices sequences which are assumed to be realisation ***x*** from some random process ***X***. The pattern-matching algorithm will then refine the *k*-tuple to groups of nearest-neighbours that are expected to reflect historical selected outcomes that better reflect future outcomes than merely the last price change or price change sequence. This is done by comparing the current realisation $x_{t}^{t-k}$ with the past.

In this way, given a set of parameters enumerating the *n*-th expert we will select the required tuple from the existing data realisations depending on the algorithm parameters using some selection function *f*

$$\begin{array}{c} x_{n,t}=x_{n(k,\ell ,w),t}=x_{k\ell w,t}=f_{\ell ,w}\left(x_{1}^{t},x_{t}^{t-k}\right) \end{array}$$ (12)

where the *m*-th component of the *k*-tuple is $x_{n,t}^{m}$.

### The log-optimal strategy

The log-optimal strategy under the assumptions of stationarity and ergodicity has been shown to be the best possible choice of strategy over the long term [1]. This type of analysis has been extended to the semi-log-optimal case [10] where weakened conditions have been derived.

The surprising result is that even with this weaker formulation the loss of optimality is such that log-optimality has, for all practical purposes, equivalent performance to portfolios selected using semi-log-optimality [10]. This provides an argument for the use of competing sequences of mean-variance portfolios in the framework of expert-based competition for capital.

With an initial investment wealth of *S*_0_ using a sequence of portfolio controls $B=\{b_{i}{\}}_{i=1}^{t-1}$ from time *i* = 1 until the current time *t* the portfolio wealth for a fully-invested portfolio is [10]

$$\begin{array}{c} S_{t}=S_{0}\Pi_{i=1}^{T}b\left(x_{1}^{i-1}\right)x_{i}^{{}_{T}}=S_{0}e^{\sum_{i=1}^{T}\text{log}\left(b\left(x_{1}^{i-1}\right)x_{i}^{{}_{T}}\right)}. \end{array}$$ (13)

This gives an average portfolio growth rate $W_{t}(B)=\frac{1}{T}\sum_{i=1}^{T}\text{log}(b(x_{1}^{i-1})x_{i}^{{}_{T}})$. The log-optimal portfolio selection problem is thus

$$\begin{array}{c} b^{*}\left(X_{1}^{t-1}\right)=\text{arg}\;\underset{b}{\text{max}}E[\text{log}(b\left(X_{1}^{t-1}\right)X_{t})|X_{1}^{t-1}]. \end{array}$$ (14)

Here one is aiming to maximize the overall wealth through the incremental selection of the sequence of fully-invested portfolio controls ***B***.

### Universally consistent strategies

The fundamental result of universal log-optimality is that no investment strategy can have a faster average rate of growth than that arising from the log-optimal portfolio [1, 2, 5, 7]. However, full knowledge of the distribution of the process is required. Strategies achieving an equivalent growth rate without knowing the distribution are called *universally consistent* [1, 10] strategies.

In principle one could via simulation enumerate all the possible controls and find via brute-force the set of controls that solve the log-optimal portfolio selection problem.

This is ambitious given current technology constraints and that the opportunity set of stocks is typically large and the data representing the features even larger—particularly for intraday quantitative trading problems.

In the idealized situation we would define some simplex Λ where there is a prior distribution *μ* on the simplex, such that some expert ***b*** is a given realisation from this distribution of portfolios. We would then directly evaluate the *μ*-weighted fully-invested universal portfolio at time *t* [5, 24]

$$\begin{array}{c} b_{t}^{*}=\frac{\int_{\Lambda }bS_{t-1}\left(b,x_{t-1}\right)d\mu \left(b\right)}{\int_{\Lambda }S_{t-1}\left(b,x_{t-1}\right)d\mu \left(b\right)} \end{array}$$ (15)

where ∫_Λ_ *dμ*(***b***) = 1 and the portfolio value *S*_*t*_ at time t is as

$$\begin{array}{c} S_{t}\left(b,x_{t}\right)=\prod_{j=1}^{t}bx_{j}^{{}_{T}}=\prod_{i=1}^{t}\sum_{j=1}^{m}b_{j}x_{j,t}. \end{array}$$ (16)

Here the portfolio is fully-invested such that ***b*1**^*T*^ = 1 for unit vector **1**.

Although we seek strategies that are universally consistent with respect to the class of stationary and ergodic processes. A pragmatic approach is required given both the unrealistic distributional assumptions, and the curse of dimensionality we face in enumerating control space (for each random process in the long-term limit the growth-rate of these strategies is equivalent to that of the log-optimal portfolio when full-knowledge of the distribution is available. In order to construct such universally equivalent strategies one needs to know the conditional distribution ***X***_*t*_ given some past $X_{1}^{t-1}$).

The strategy is to reduce the problem by finding a more informed subset of controls that can be used to approximate the required sequence of portfolio controls that are used to represent a universally consistent strategy. In addition to reducing the set of applicable controls one also aims to streamline the evaluation of these controls and their adaption through time, this can be achieved by reducing the log-optimality criterion to semi-log-optimality.

### Semi-log optimality

We choose to focus on the first two moments of the price relative distributions: the mean and covariance. This will allow enhanced performance speed of the algorithms (see Intraday JSE Data section) but with some loss in long-term optimality [10, 25] and as such a deviation from the universally consistent strategies.

First, we have reduced the opportunity space in the simplex of all possible portfolios in order to make the problem of finding a portfolio that is optimal over the entire feature space computationally tractable, this is achieve by using expert-generating algorithms and learning over the free-parameters for those experts generating algorithms.

Second, we replaced the optimization with a quadratic approximation that will give us analytic solutions to replace optimizations that we would otherwise have to solve numerically. In addition to a performance advantage, using the quadratic approximation this will also provide a straight-forward method for considering both fully-invested and zero-cost portfolio’s in a single framework.

Streamlining the algorithms for performance was approached in two steps, first, to separate the problem into that of an online-learning algorithm and the expert generating algorithms, then, second, to reduce the log-optimality criterion to semi-log-optimality.

The semi-log-optimal portfolio selection takes on the form

$$\begin{array}{c} b^{*}\left(X_{1}^{t-1}\right)=\text{arg}\;\underset{b}{\text{max}}E[h(b\left(X_{1}^{t-1}\right)X_{t})|X_{1}^{t-1}]. \end{array}$$ (17)

where $h(z)=(z-1)-\frac{1}{2}(z-1{)}^{2}$ from the second order Taylor expansion of log(*z*) at *z* = 1.

A related approach was taken in [24] where they derived an analytic approximation for an efficient universal portfolio. Our simplified mean-variance approach was motivated by their development of an analytic algorithm, the difference here is that we want an algorithm that is online, analytic, explicitly includes zero-cost portfolios, and allows for the restriction of the solution space using some expert generating algorithm directly at each step rather than via side-information.

### Active fund separation problem

The determination of the optimal portfolio is sequentially implemented using the exact solution to the quadratic approximation to log-optimality by solving the active fund selection problem. The active fund selection problem is a special case of the mutual fund selection problem [19, 20]. This will give an analytic approximation that can both cater for long-only fully-invested experts (absolute experts) as well as leverage one (∑_*i*_|*ω*_*i*_| = 1 for portfolio controls *ω*) zero-cost portfolio’s (active experts).

We therefore consider the semi-log-optimal portfolio optimization problem [19, 20, 26] for return expectation vector ***μ*** and asset return covariance matrix Σ with a portfolio control vector ***ω*** in terms of the risk aversion parameter *γ*. The conjugate transpose of a vector is denote as (⋅)^*T*^ over a single investment period to define the control problem as:

$$\begin{array}{c} \underset{\omega }{\text{max}}\{\omega^{{}_{T}}\mu -\frac{\gamma }{2}\omega^{{}_{T}}\Sigma \omega \}\;\text{s.t.}\;\omega^{{}_{T}}1=1. \end{array}$$ (18)

Here we have changed notation to denote the portfolio controls as ***ω*** in order to avoid confusion with the portfolio strategy controls ***b*** that are the result of the online-learning algorithm which aims to approximate the semi-log-optimal portfolio selection strategy for aggregate portfolio controls ***b***_*t*_ for time increment *t*.

Here the portfolio controls ***ω*** are used to generate the experts that populate the expert control set ***H***_*n*,*t*_. It is the expert control set that is then used to generate the semi-log-optimal portfolio choice at each time *t*: ***b***_*t*_.

Eq (18) can be rewritten as the mutual-fund Lagrangian

$$\begin{array}{c} L=\omega^{{}_{T}}\mu -\frac{\gamma }{2}\omega^{{}_{T}}\Sigma \omega -\lambda_{\omega }\left(\omega^{{}_{T}}\;1-1\right). \end{array}$$ (19)

and solved using elementary Kuhn-Tucker methods. Two equations are found in terms of the optimal solution for the portfolio control, ω*, the first gives the quadratic optimal risk-return pay-off, and the second, the fully-invested portfolio investment constraint

$$\omega^{*}=\frac{1}{\gamma }\Sigma^{-1}(\mu -\lambda_{\omega }1),$$ (20)

$$\omega^{{*}_{T}}1=1.$$ (21)

The Lagrange multiplier is determined by substituting Eq (20) into Eq (21) to find:

$$\begin{array}{c} \lambda_{\omega }=\frac{1^{{}_{T}}\Sigma^{-1}\mu }{1^{{}_{T}}\Sigma^{-1}1}-\frac{\gamma }{1\Sigma^{-1}1}. \end{array}$$ (22)

This is then used to eliminate the Lagrange multiplier from Eq (20) to find a formulation of the mutual fund separation theorem:

$$\begin{array}{c} \omega^{*}=\frac{\Sigma^{-1}1}{1^{{}_{T}}\Sigma^{-1}1}+\frac{1}{\gamma }\Sigma^{-1}(\mu -1\frac{1^{{}_{T}}\Sigma^{-1}\mu }{1^{{}_{T}}\Sigma^{-1}1}). \end{array}$$ (23)

The first term on the right is the lowest risk portfolio and the second term is the zero-cost portfolio that encapsulates the relative views of the assets. We will typically work with the separation theorem in the form given in Eq (23). The second term will give us an efficient method of generating zero-cost portfolio’s.

It is then convenient to re-write the Mutual Fund Separation theorem to an Active Fund Separation theorem explicitly from Eq (23) by defining the lowest risk portfolio as the benchmark portfolio:

$$\begin{array}{c} \omega^{*}=\omega_{{}_{B}}+\omega_{{}_{A}}, \end{array}$$ (24)

where

$$\omega_{{}_{B}}=\frac{\Sigma^{-1}1}{1^{{}_{T}}\Sigma^{-1}1},$$ (25)

$$\omega_{{}_{A}}=\frac{\Sigma^{-1}}{\gamma }(\frac{\mu 1^{{}_{T}}-1\mu^{{}_{T}}}{1{}_{T}\Sigma^{-1}1})\Sigma^{-1}1,$$ (26)

The formulae for ***ω***_*B*_ and ***ω***_*A*_ will be directly used in the expert generating algorithms based on views encoded in the mean, ***μ***, and the covariances, Σ, as a function of the various expert generating parameters. The resulting controls ***H***_*n*,*t*_ will then be determined from the *m*-th component of either ***ω***_*A*_ for the active experts or ***ω***_*B*_ + ***ω***_*A*_ for the absolute experts for the *n*-th expert for time-increment *t*.

For situations where we want experts constructed from zero-cost portfolios we will use the tactical solution from Eq (26) to generate the experts for a given *k*-tuple. In situations where we need fully invested experts we will use the combination of the benchmark fund and the active (or tactical) fund.

Suppressing indexes over the *m* objects the expert controls for the *n*-that expert for the two possible cases: (1.) the absolute experts, and (2.) the active experts is then

$$\begin{array}{c} H_{n,t}=\{\begin{array}{c} h^{{}_{T}}1=1,h=\omega_{{}_{B}}\left(\Sigma \right)+\omega_{{}_{A}}\left(\gamma ,\mu ,\Sigma \right)\;\text{s.t.}\;h\geq 0\\ h^{{}_{T}}1=0,h=\omega_{{}_{A}}\left(\gamma ,\mu ,\Sigma \right)\;\text{s.t.}\;\sum |h|=1\Leftrightarrow {\left|h\right|}^{{}_{T}}1=1 \end{array} \end{array}$$ (27)

Here the *m*-th component of ***H***_*n*,*t*_ is *H*_*nm*,*t*_ and the portfolio weights are dependent on the expert-tuples ***x***_*n*,*t*_ for a given expert

$$\omega_{{}_{A}}=\omega_{{}_{A}}\left(\gamma ,\mu \left(x_{n,t}\right),\Sigma \left(x_{n,t}\right)\right)$$ (28)

$$\omega_{{}_{B}}=\omega_{{}_{B}}\left(\Sigma \left(x_{n,t}\right)\right).$$ (29)

For the active expert we enforce the leverage unity constraint at the beginning of each time increment, this can be considered equivalent to setting the risk-aversion *γ*, at the beginning of each time increment, such that the leverage is always unity.

This is an important feature of the algorithm as we do not enforce uniform risk-aversion through time. We rather choose to ensure that capital be fully utilized given the available information. The following sections describe how the expert-tuples are constructed for the various expert generating algorithms.

### Expert generating algorithms from patterns

In order to efficiently reduce the space of portfolio controls to efficiently generate a reasonable approximation to universally consistent strategies using Eq (15) we reduce the set of applicable controls using expert-generating algorithms. The expert-generating algorithm we use in our numerical experiments will be a pattern-matching algorithm [10]. One can make various decisions about how to break data up into manageable pieces for the various algorithms, the most basic decisions relate to how to break up the data in time, we call this partitioning, the other choice relates to how we break the data up in terms of the objects themselves (often called the features), this we call clustering. Partitioning is typically a more intricate task because this has implications for the algorithm and system structure.

The pattern-matching algorithm is based on two steps subsequent to the choice of clusters *s*(*n*): (1.) partitioning and (2.) pattern-matching. Clusters can be chosen by a variety of methods, we would like to promote two methods: (i) correlation matrix based methods [27], and (ii) clusters based on economic classifications of stocks (for example, using ICB (Industry Classification Benchmark) sectors classifications [28]). The prior method, correlation based methods, have outputs that can be directly used as inputs into the algorithms discussed here, specifically via *s*(*n*), the cluster membership parameters. It is however, the method based on fixed economic sector classifications [28], that will be explicitly used in this paper for the intraday experiments in the Intraday JSE Data section, this is both for speed and simplicity. It should be noted that using ICB sectors to generate additional experts for the daily simulations does boost algorithm wealth performance but we chose to explicitly demonstrate the value of including sector information in the context of the intraday strategies.

In the daily numerical experiments we have ignored the impact of clustering and used the clusters *s*(*n*) of the *n*-th stock as being trivial, *i.e.* we consider a single stock cluster that includes all *m* objects. The inclusion of cluster indexing can be important to the practical implementation of these techniques as it is often useful to restrict trading signal decisions to similar stocks. There is a wealth advantage to this, as we have shown when we considered the impact of clustering for the numerical experiments using intraday data (see Intraday JSE Data section).

The pattern matching algorithm is split into two key components: First, the *partitioning algorithm*, which selects a collection of time-ordered features from the full set of feature data. Second, the *pattern-matching algorithm*, where a given measured pattern is extracted from the feature data and used by the algorithm to find similar patterns, in a given partition, elsewhere in the feature data.

#### Partitioning

Subsets of time-ordered data are selected from the original time-order data for a given collection of objects. The collection of objects can in turn be a sub-collection of the original set of objects. Partitioning takes place in the time domain while clustering is in the object dimension. The purpose of partitioning is to prepare data subsets for pattern-matching [4]. Four distinct approaches to data partitioning are enumerated here, however only the trivial partition is used in the experiments.

A partition is a collection {*p*_*t*_}_*ℓ*_ represented by a logical vector of the length of a given time-series where true is represented as one and false as zero to index membership in a given partition. When a partition is determined from features that determine the state of the system at a given time we will use that partition to represent the system in that state for the sake of pattern-matching.

For the numerical experiments presented here we will use variations of the *trivial partition*: Here all the temporally ordered data is kept in a single partition as represented by a vector of ones of length of the time-series.

$${\left\{p_{t}\right\}}_{1}=\left\{\left(1,\ldots ,1,1,1\right)\right\}.$$ (30)

There are wealth advantages associated with more sophisticated partitions. We considered four different partitioning approaches: the *trivial partition*, the *over-lapping partition* (Eq (31) shows an example of length T overlapping partition of features): were data membership in partitions is repeated in order to bias the data towards a given time, for example, the last time-increments is repeated across all *ℓ* partitions for time-series of length T, the *exclusive partition* where the partitions are mutually exclusive subsets of the full partition, and the *side-information partition* [5].

$${\left\{p_{t}\right\}}_{T}=\{(0,\ldots ,0,0,1),(0,\ldots ,0,1,1),\ldots ,(1,\ldots ,1,1,1)\}.$$ (31)

The most heuristically useful partition is that of the *side-information partition* where partitions can be pre-selected in the partitioning algorithm based on rules conditioned on side-information [5], partitioning can be both useful as a nuanced exploitation of information, for example by splitting feature data over different regimes, and thus to generate distinct experts for different regimes, and as an effective approach to parallelization of algorithms.

Here we would partition the time-series based on side-information arising from additional features drawn from the system being observed as in [5]. For example, we could use a Markov-switching algorithm with *ℓ* states, assign each time in the time-series a state index and the define the partition membership based on states, or we could choose a feature as side-information and *ℓ*-tile the data into *ℓ* groups and then based on whether a given time has a side-information feature in a particular group it would be assigned to a given partition.

Partitioning serves as a convenient mechanism for breaking up the feature data into distinct states. This can be useful when choosing to search for patterns when the system is in a distinct state as it will enable the algorithm to search for patterns only in historic data residing from times in the past when the system was in a similar state. By combining a partitioning algorithm with a state-detection algorithm one can both improve computational times as well as algorithm performance in terms of wealth generation [29], this is not explored further here.

#### Pattern-matching

The pattern-matching algorithm will take a *k*-tuple and search a given partition of the feature data (see the algorithm in S2 Appendix) for similar patterns by finding the smallest distance measure between the *k*-tuple and data in a given partition using a matching algorithm (see S3 Appendix). This best matching set of data in the partition will then be used to determine a pattern-matching time *j*_*ℓ*_. The matching time will then be used to select a future outcome some time period *τ* ahead of the matched pattern. This future outcome is used to construct a tuple of data, the expert-tuple, iteratively using the look-ahead rule: *j*_*n*_ = *j*_*ℓ*_ + *τ*. A number of such pattern-matches will be accumulated to construct the expert-tuple ***x***_*n*,*t*_ and from this a mean and covariance are computed.

This mean and covariance will then serve as the input into Eq (24) to determine that expert controls ***H***_*n*,*t*+1_, the *n*-th experts controls to be held for time-period *t* + 1.

The pattern-matching algorithm is split into two separate algorithms. The first algorithm, which we will call the pattern algorithm, generates patterns to be matched and partitions of data into which the pattern will be matched. The second algorithm will then take the pattern and the data partitions and generate matching times. The matching times will then be used to generate an expert-tuple ***x***_*n*,*t*_.

The pattern algorithm generates a *k*-tuples $\{x_{t-k}^{t}{\}}_{s(n)}$ [4] for matching, and a data partition {***x***_*t*_}_(*p*_*ℓ*_,*s*(*n*))_ using a predefined temporal partition {*p*_*ℓ*_} of the data and the cross-sectional cluster for the *n*-th expert *s*(*n*). This is iteratively done for each expert as enumerated by the parameters that define a given expert: the cluster membership *w* = *s*(*n*) of the *n*-th expert, the partition variable *ℓ*, the *k*-tuple variable *k* and the look-ahead horizon variable *τ*.

For each set of variables that define the *n*-th expert the pattern algorithm will then call the matching algorithm.

The matching algorithm (see S3 Appendix) will find matches for the *k*-tuples, $x_{t-k}^{t}$ in the partitions. This is used by the pattern-matching algorithm (see S2 Appendix) which selects a *k*-tuple that is to used by the matching algorithm. If there is a single partition of data, the matching algorithm will find the $\hat{\ell }$ closest matches. We consider two rules for calculating $\hat{\ell }$ and will refer to these as rule *P*. This rule is introduced in order to easily compare our algorithms with prior literature, more specifically [4, 10]. The difference is related to how the partitions are defined and implemented.

We consider the *trivial rule*: $\hat{\ell }=\ell$ and the rule required to recover the Nearest-Neighbour (NN) algorithm performance described in [4]. The *Györfi et al Nearest Neighbour rule* is where $\hat{\ell }$ is determined by a variable *p*_*ℓ*_ ∈ (0,1). The choice of *p*_*ℓ*_ used in the experiments is the same as in [4].

$$\hat{\ell }=\lfloor p_{\ell }t\rfloor \;\;\text{where}\;\;p_{\ell }=0.02+0.5\frac{\ell -1}{L-1}$$ (32)

Here *t* represents the number of time periods in the history, and the floor is taken to find the smallest partition at the given time. This modification serves primarily to allow us to recover prior results in the literature using the framework we implemented in the software for the numerical experiments.

If there are *ℓ* partitions of data the algorithm will find the best match in each partition. The matching algorithm will find *ℓ* best matches and from those best matches extract *ℓ* matching times *j*_*ℓ*_ associated with the time of each *k*-tuple match. From the look-ahead rule the matching algorithm will then construct the expert-tuple ***x***_*n*,*t*_. The matching algorithm will then compute the expert-control for this given expert-tuple ***h***_*n*,*t*_.

The distance between tuples is the 2-norm. Although we could use the distance between two matrices as the general distance in the algorithm, we have chosen to differentiate selecting the most recent vectors of object features and the test-tuple as the vector distance between these two vectors only for the case of *k* = 1, while for *k* > 1 we measure the distance of each object from the same object at a different time independently from other objects.

This will rather allow us to search for the best fits of objects independently rather than in collective. This is an important refinement, in the original version of the algorithm we followed [4] and used the 2-norm in full generality independent of the window size *k* we found better performance by independently selecting for patterns using column-wise computed distances.

## Data description

### OHLC data

The data we will consider will be sequential data, but not necessarily continuously sequential. For this reason we will study OHLC (Open-High-Low-Close) bar-data where the closing price of a given bar is not necessarily the opening price of the subsequent bar of the data. We will first study daily sampled data and then intraday data. The algorithms will be initially tested using synthetic data (see Synthetic Data section), and then the real world test data used in prior research [2, 4] (see Real Data section) which are sequences of daily sampled closing prices.

The data and algorithms can be easily extended to accommodate additional features as side-information [5]; such as volumes, spreads, and various financial indicators and asset specific and state attributes. The side-information can be trivially used to re-partition data into additional sets of experts and then used as inputs into the learning algorithm. The wealth performance enhancement relating to the side-information extension is not demonstrated in the numerical experiments presented here.

**Fig 1:**
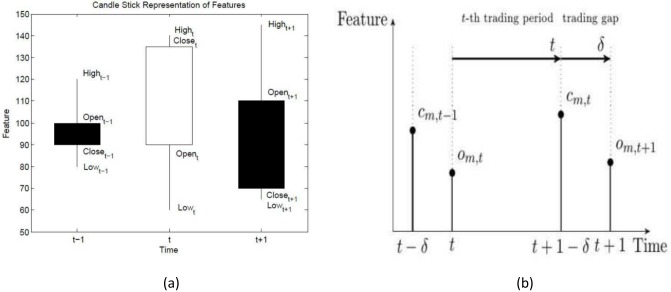
Feature time-series data. (a) The feature time-series data is best thought of as OHLC (Open-High-Low-Close) bar data. The filled box in the candle chart denotes the situation where the close price is lower than the open price, conversely the unfilled box has the close price higher than the open price. (b) Feature time-series investment period for the *t*-th time increment showing that the end of the *t*-th increment does not always have to coincide with the start of the next, here the *t*+1-th, investment period. The opening price is denote as *o*_*m*,*t*_ and the close price for the period as *c*_*m*,*t*_ for the *m*-th asset.

OHLC bar data is typically represented by a candle-stick graph as in Fig (1).

The time-series data is such that the closing price of time-increment *t* is not necessarily at time *t* + 1 the start of time increment *t* + 1. The closing price can in fact be at some time *t* + *δ* for some arbitrary data-specific time-increment *δ*.

A low-frequency example is that of a typical trading day on the JSE, the market opens in the morning with some opening price, *o*_*t*_, at 9h00, the market may then close at some closing time 17h00, after a closing auction period, the official closing price *c*_*t*_, is then printed soon after the market close (perhaps after some randomisation period). The market is then closed for some time-period over-night until the market opens again on the subsequent day. There is a period, *δ*, when the market is closed and as such information is not continuously being priced into the traded assets. Information that accumulates over-night will then be priced into the market prices through the process of the opening auction and subsequent trading in the various assets.

Our approach to OHLC data is applicable to a variety of synchronously sampled or re-sampled data sets, including intraday data:

1. *close-to-close*: Here the prices *p*_*m*,*t*_ for the *m*-th assets are the time-series of close prices. The price relatives *x*_*m*,*t*_ are then the computed from the close price time-series *c*_*m*,*t*_
  $$x_{m,t}=\frac{c_{m,t}}{c_{m,t-1}}.$$ (33)
  The algorithm is trying to exploit information relating to price changes from the close of trading of one time increment to the close of trading of a subsequent time increment.
2. *open-to-close*: Here the prices *p*_*m*,*t*_ for the *m*-th assets are the ordered time-series pairs of open and close prices on the same data the price relatives are then computed as
  $$x_{m,t}=\frac{c_{m,t}}{o_{m,t}}.$$ (34)
  Here one is trying to exploit price relative changes within a trade increment, for example, across a single day from the market opening to the market close ignoring the over-night price changes.
3. *close-to-open*: Here the prices *p*_*m*,*t*_ for the *m*-th asset are the price changes from the close of the trade period at *t* − 1 to the next trade period at time *t*
  $$x_{m,t}=\frac{o_{m,t}}{c_{m,t-1}}.$$ (35)
  Here one is looking to exploit the change in prices between trade periods where the information cannot yet be fully reflected in trading until the trading commences in the next trade period.
4. *open-to-open*: Here the prices *p*_*m*,*t*_ for the *m*-th asset are the time-series of opening prices. The price relatives *x*_*m*,*t*_ are then computed from the opening prices *o*_*m*,*t*_
  $$x_{m,t}=\frac{o_{m,t}}{o_{m,t-1}}.$$ (36)
  This is looking for inefficiencies in the prices changes from market opening to market opening.

The important missing component of information is that related to volume (and additional features such as spread, order-imbalance and order-book resilience for intraday data). For example, the opening price is a less reliable price when it has been determined off significantly lower volumes of trading, as compared with a typical closing price. In the case where the closing auction of a given market has more volume than the typical opening auction the relative uncertainties in the prices can be substantial. The typical time increment for a given feature is given in Fig (1). We promote the use of a state-detection algorithm and side-information partitioning in order to address these types of concerns. In the context of this work such issues do not change our conclusions.

It is expected that the learning algorithm will still attempt to maximise the long-term wealth given a specific expert generating algorithm for a given feature set. For both daily data and intraday data the feature set that is of most interest to us in this study will be those associated with the “close-to-close” and “close-to-open” price relative features.

### Synthetic data

The algorithm was tested on four synthetic data cases (SDC) for both active and absolute portfolios. The synthetic data was generated for 10 stocks over 1000 time periods. The price relatives *x*_*m*,*t*_ for each stock at each time period was randomly generated from a lognormal distribution (lognrnd function in MATLAB generated using the Mersenne Twister psuedorandom number generator [30] and initialised using a specific seed value), each synthetic data case defines a mean, *μ*, and variance, *v*, used to generate the dataset. The mean, $\bar{\mu }$, and standard deviation, $\bar{\sigma }$, of the associated normal distribution is given by:

$$\bar{\mu }=\text{log}(\frac{\mu^{2}}{\sqrt{v+\mu^{2}}})$$ (37)

$$\bar{\sigma }=\sqrt{\text{log}(\frac{v}{\mu^{2}}+1)}$$ (38)

Table 1 summarises the four synthetic data cases, each case was generated 30 times and initialised with seed values 1, 2, …, 30 respectively.

1. **Synthetic Data Case 1 (SDC 1):** was generated from a lognormal distribution with a mean price relative, *μ* = 1, and a variance, *v* = 0.0002, to simulate a stock market where there is no significant increase or decrease in the value of a stock over time.
  The expected outcome is that neither the active portfolio nor the absolute portfolio will be able to learn which stocks it should hold a long position or short position.
2. **Synthetic Data Case 2 (SDC 2):** was generated from a lognormal distribution with a mean price relative, *μ* = 1.001, and a variance, *v* = 0.0002, to simulate a stock market where the value of the stocks are increasing over time.
  The expected outcome is that the absolute portfolio will learn which stocks to hold a long position on, however the active portfolio will not be able to learn which stocks to hold a short position on as no stocks decrease in value over time.
3. **Synthetic Data Case 3 (SDC 3):** was generated from a lognormal distribution with a random mean price relative, *μ* ≥ 1, assigned to each stock and a variance, *v* = 0.0002. The random means is calculated as follows:
  $$\mu_{m}=1+\max\left[0,\min\left(0.0005+0.0005\delta ,0.001\right)\right]$$ (39)
  where *δ* is a random number generated from a standard normal distribution (using the randn function in MATLAB with the Mersenne Twister psuedorandom number generator [30] and initialised using a specific seed value). This simulates a stock market where some stocks are increasing in value and some stocks are decreasing in value over time.
  The expected outcome is that both the active portfolio and the absolute portfolio will learn to hold a long position on the stocks increasing in value over time and hold a short position on the stocks decreasing in value over time, however it is expected that the absolute portfolio will beat the active portfolio due to the growth rate of the stocks increasing in value over time.
4. **Synthetic Data Case 4 (SDC 4):** was generated from a lognormal distribution with mixed means assigned to the price relatives, *μ* = 0.999 was assigned to 3 stocks and *μ* = 1.001 was assigned to the remaining stocks, and a variance, *v* = 0.0002. This dataset simulates a stock market where the value of some stocks are increasing and the value of some stocks are decreasing.
  The expected outcome is that both the active portfolio and the absolute portfolio will learn to hold a long position on the stocks increasing in value over time and hold a short position on the stocks decreasing in value over time.

**Table 1:** Summary of random datasets.

Dataset	*μ*	*v*
SDC 1	1.000	0.0002
SDC 2	1.001	0.0002
SDC 3	random≥ 1	0.0002
SDC 4	mixed	0.0002

The means and variances that were chosen when generating the synthetic data sets. The random means for SDC 3 was calculated using Eq (39) and the means for SDC 4 was generated as described in the Synthetic Data section.

### Real data

The algorithm is tested on four sets of real data, summarised in Table 2, two data sets from the New York Stock Exchange (NYSE) obtained at [21] and two data sets from the Johannesburg Stock Exchange (JSE) obtained at [31].

1. **NYSE Data:** This is described in [21] and contains *close-to-close* price relatives for 36 stocks listed on the New York Stock Exchange from 1962-1984. This is the same data set used in [4, 10] and [2].
2. **NYSE Merged Data:** This is described in [21] and the dataset contains *close-to-close* price relatives data for 23 stocks listed on the New York Stock Exchange from 1962-2006. The data of the 23 stocks during 1962-1984 is identical to the data described above in point 1.
3. **JSE OHLC Data:** This was obtained from Thomson Reuters Tick History (TRTH) [31] and contains daily data for 42 stocks listed on the Johannesburg Stock Exchange (JSE) from 1995-2015 (using RIC chain 0#.JTOPI), however not all of the 42 stocks were listed in 1995 and the data for these stocks begins at a later time (The data has an implicit survivorship bias however this does not impact the results of this paper). The data lists the open, high, low and close prices for all of the 42 stocks. This raw data was processed into four datasets containing *close-to-close*, *open-to-close*, *close-to-open* and *open-to-open* price relatives respectively. Splits, mergers and missing data were handled by assigning a price relative of 1 for that day. Splits and mergers were identified as having a *x*_*m*,*t*_ < 0.7 and *x*_*m*,*t*_ > 1.3 respectively.
4. **JSE Intraday Data:** The transaction data was obtained from Thomson Reuters Tick History (TRTH) [31] and consisted of top-of-book and transaction updates for 40 stocks listed on the Johannesburg Stock Exchange (JSE) during 2013 in RIC chain 0#.JTOPI. The transaction data was converted into 5-minute bar data using the trade price and volume weighted averaging. The 5-minute bar-data starts at 9h30 and ends at 16h30 for normal trading days and starts at 9h30 and ends at 11h30 for early close days. A normal trading data on the JSE starts with an opening auction between 8h30 and 9h00, continuous trading takes place between 9h00 and 16h50, and the day ends with a closing auction between 16h50 and 17h00.

**Table 2:** Summary of real datasets.

Data Set	Time Period	# Stocks
NYSE [21]	1962-1984	36
NYSE Merged [21]	1962-2005	23
JSE daily OHLC [31]	1995-2015	42
JSE Intraday [31]	2013	40

Description of the real data sets that the algorithm was tested on.

## Implementation

The wealth achieved by the portfolio and the wealth achieved by the experts is determined using the Online Learning Algorithm (OLA) (See S1 Appendix). The expert controls ***H***_*n*,*t*_, introduced in the section An Online-Learning Algorithm for Portfolio Selection, used in the Online Learning Algorithm are determined by using the Pattern Matching Algorithm (PMA) (see S2 Appendix), which calls the Matching Algorithm (MTA) to determine the expert controls for each expert. The Matching Algorithm (see S3 Appendix) updates an experts’ wealth as described in Eq (27). In the experiments 50 experts were used with *K* = (1, 2, …, 5) and *L* = (1, 2, …, 10), similar to choice of experts used in [4, 10].

All Results and data processing was done in MATLAB. The algorithm was implemented for both the absolute and active case using a MATLAB class that we named *pattern*, a MATLAB class was used instead of function because this allows the algorithm to easily be extended to a more online approach. The *pattern* class was extended to include our recovered version of the Györfi *et al* Nearest Neighbour [4] algorithm so that the running time comparisons in the Results and Analysis section will be accurate. The Cover *et al* [2] Universal Portfolios algorithm was recovered by creating a MATLAB function that implement the algorithm.

## Results and analysis

### Synthetic data

The algorithm was tested on four synthetic data cases (SDC) to illustrate how the algorithm performs in different types of markets. Table 3 displays the best and average wealth achieved by the active and absolute portfolios for 30 runs of each synthetic data case initialised with seed values 1,2, …, 30 respectively.

**Table 3:** Wealth (*S*) from investing in synthetic data.

Data	Port.	Wealth		Best Expert	
		Best	Avg.	Best	Avg.
SDC 1	Abs.	1.231	0.992	1.806	1.250
	Act.	1.451	1.052	1.753	1.358
SDC 2	Abs.	3.241	2.612	4.654	3.270
	Act.	1.451	1.052	1.753	1.358
SDC 3	Abs.	2.320	1.685	3.091	2.090
	Act.	1.490	1.171	1.782	1.451
SDC 4	Abs.	2.455	1.896	2.931	2.250
	Act.	2.927	2.055	3.270	2.297

Wealth achieved by the active and absolute portfolios for 30 runs of each synthetic data case.

**Fig 2:**
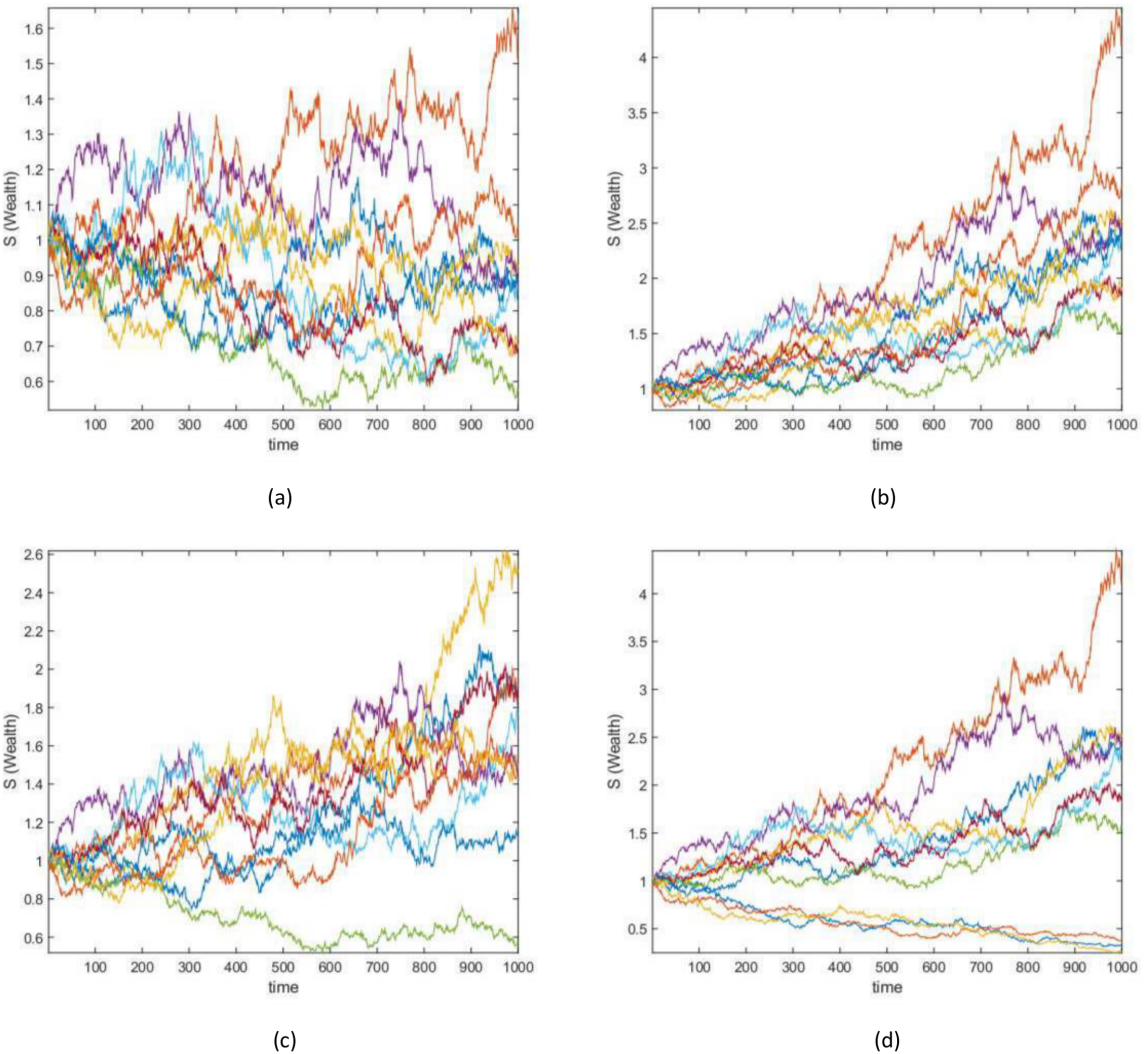
Synthetic data case wealth gained. The wealth achieved by each randomly generated stock for (a) SDC 1 (b) SDC 2 (c) SDC 3 and (d) SDC 4.

**Fig 3:**
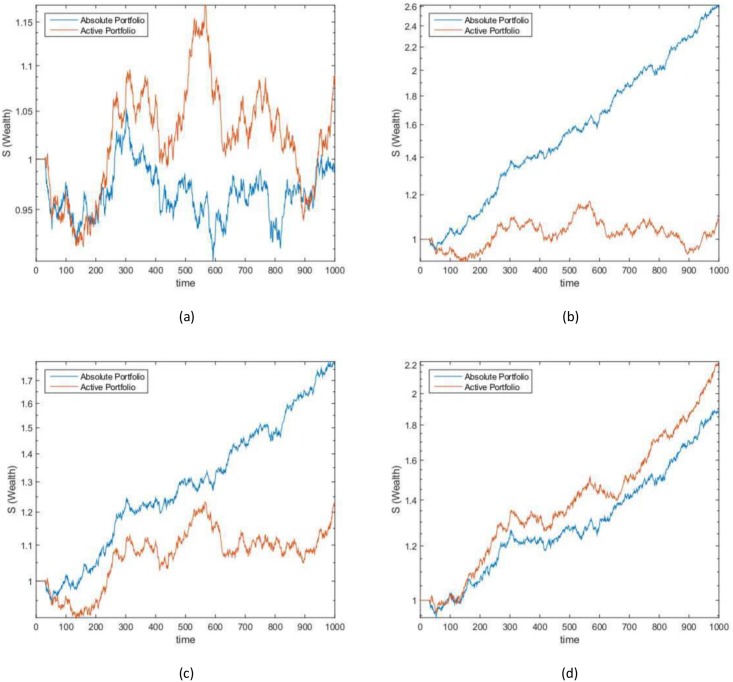
Synthetic data case wealth gained. The wealth achieved by the active and absolute portfolios on (a) SDC 1 (b) SDC 2 (c) SDC 3 and (d) SDC 4 that consists of a time period of 1000 and 10 stocks.

On all of the datasets the algorithm, when using absolute portfolio, eventually learns the stocks that are increasing in value over time; this is observed from SDC 2, 3 and 4. Similarly, the algorithm, when using active portfolio, eventually learns to hold a long position on the stocks that are increasing in value over time and hold a short position on the stocks that are decreasing in value over time; as observed for SDC 3 and 4. Fig 2 shows the wealth achieved by each synthetic stock when randomly generated using an initial seed value of 7, Fig 3 shows the wealth achieved by the active and absolute portfolios.

Tables 4 and 5 display average *p* values from the two-sample Kolmogorov-Smirnov tests when comparing the following combinations of the total wealth gained from the portfolio (*S*_1_), the wealth gained from the best expert of the portfolio (*S*_2_) and the wealth gained from the best stock (*S*_3_):

1. *S*_2_ > *S*_1_: The alternative hypothesis that the cumulative distribution function (CDF) of the wealth gained from the best expert of the portfolio, *S*_2_, is larger than the CDF of the total wealth gained from the portfolio, *S*_1_, at the 5% significance level.
2. *S*_2_ > *S*_3_: The alternative hypothesis that the CDF of the wealth gained from the best expert of the portfolio, *S*_2_, is larger than the CDF of the wealth gained from the best stock, *S*_3_, at the 5% significance level.
3. *S*_3_ > *S*_1_: The alternative hypothesis that the CDF of the wealth gained from the best stock, *S*_3_, is larger than the CDF of the total wealth gained from the portfolio, *S*_1_, at the 5% significance level.

**Table 4:** Average *p* values of wealth (*S*) for active portfolios.

	Best Expert vs. Tot. Wealth		Best Expert vs. Best Stock		Best Stock vs. Tot. Wealth	
Hyp.	*S*_2_ > *S*_1_		*S*_2_ > *S*_3_		*S*_3_ > *S*_1_	
	$\bar{p}$	$p>\bar{p}$	$\bar{p}$	$p>\bar{p}$	$\bar{p}$	$p>\bar{p}$
SDC 1	0.809	0.172	0.031	0.000	0.654	0.013
SDC 2	0.809	0.172	0.000	0.000	0.873	0.407
SDC 3	0.830	0.172	0.000	0.000	0.904	0.563
SDC 4	0.725	0.013	0.000	0.000	0.622	0.006

Comparisons of the average *p* values of the wealth gained from the active portfolio. The first *p* value in each column is average *p* value, of the 30 data sets for each case, using two-sample Kolmogorov-Smirnov tests for the alternative hypotheses (Hyp.). The second *p* value is obtained from the two-sample Kolmogorov-Smirnov tests for the alternative hypothesis that the cumulative distribution function (CDF) of the *p* values for the 30 data sets for each case is larger than the CDF of the average *p* value at the 5% significance level.

**Table 5:** Average *p* values of wealth gained (*S*) from the absolute portfolio.

	Best Expert vs. Wealth		Best Expert vs. Best Stock		Best Stock vs. Wealth	
Hyp.	*S*_2_ > *S*_1_		*S*_2_ > *S*_3_		*S*_3_ > *S*_1_	
	$\bar{p}$	$p>\bar{p}$	$\bar{p}$	$p>\bar{p}$	$\bar{p}$	$p>\bar{p}$
SDC 1	0.893	0.407	0.000	0.000	0.647	0.013
SDC 2	0.642	0.013	0.000	0.000	0.567	0.001
SDC 3	0.593	0.002	0.000	0.000	0.795	0.100
SDC 4	0.846	0.274	0.000	0.000	0.644	0.006

Comparisons of the average *p* values of the wealth gained from the absolute portfolio. The first *p* value in each column is the average *p* value, of the 30 data sets for each case, using two-sample Kolmogorov-Smirnov tests for the alternative hypotheses (Hyp.). The second *p* value is obtained from the two-sample Kolmogorov-Smirnov tests for the alternative hypothesis that the cumulative distribution function (CDF) of the *p* values for the 30 data sets for each case is larger than the CDF of the average *p* value at the 5% significance level.

The two-sample Kolmogorov-Smirnov test was chosen because it is a non-parametric test and makes no assumption about the distribution of the datasets.

Tables 6 and 7 display average *p* values from the two-sample Kolmogorov-Smirnov tests when comparing wealth gained from the datasets. It can be observed that the active portfolio performs the best on SDC 4 and the absolute portfolio performs the best on SDC 2.

**Table 6:** Comparison of *p* values of wealth gained (*S*) from the active portfolio.

	**SDC 1**	**SDC 2**	**SDC 3**	**SDC 4**
**SDC 1**	-	0.9765	0.0669	0
**SDC 2**	0.9786	-	0.0669	0
**SDC 3**	0.7928	0.7916	-	0
**SDC 4**	0.9653	0.9649	0.9535	-

Comparison of the average *p* values from two-sample Kolmogorov-Smirnov tests for the alternative hypothesis that the cumulative distribution function (CDF) of wealth gained from the active portfolio on SDC *i* is larger than the CDF of wealth gained from the active portfolio on SDC *j* at the 5% significance level, where *i* represents the rows and *j* represents the columns of the table. The *p* values is the average of 30 comparisons, each comparison using a seed value of 1, 2, …, 30 respectively.

**Table 7:** Comparison of *p* values of wealth gained (*S*) from the absolute portfolio.

	**SDC 1**	**SDC 2**	**SDC 3**	**SDC 4**
**SDC 1**	-	0	0	0
**SDC 2**	1.0000	-	1.0000	0.9997
**SDC 3**	1.0000	0	-	0.1289
**SDC 4**	1.0000	0	0.5055	-

Comparison of the average *p* values from two-sample Kolmogorov-Smirnov tests for the alternative hypothesis that the cumulative distribution function (CDF) of wealth gained from the absolute portfolio on SDC *i* is larger than the CDF of wealth gained from the absolute portfolio on SDC *j* at the 5% significance level, where *i* represents the rows and *j* represents the columns of the table. The *p* values is the average of 30 comparisons, each comparison using a seed value of 1, 2, …, 30 respectively.

### NYSE data

**Fig 4:**
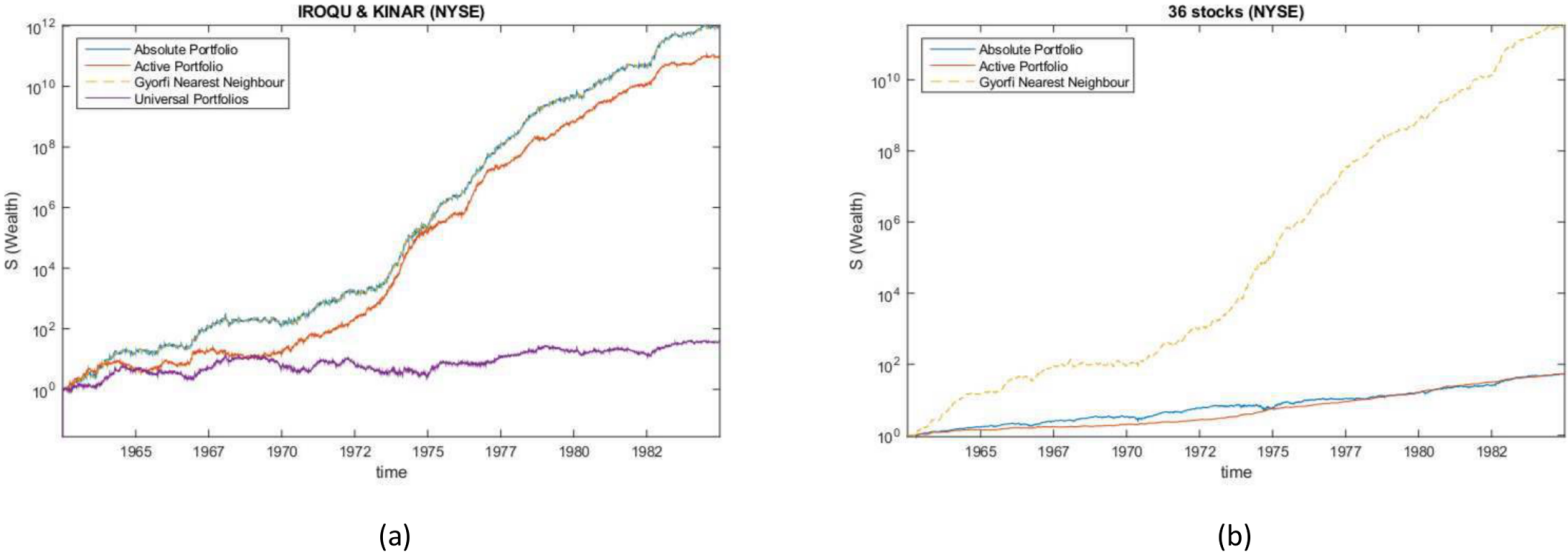
Wealth gained in NYSE data experiments. Comparison of the wealth gained from different methods when investing in (a) iroqu and kinar (b) 36 stocks from the NYSE dataset.

The algorithm was run on the NYSE data set for both absolute and active portfolios on the same pairs of stocks used by [2] and by [4, 10]. Table 8 and Fig 4 shows the wealth achieved by the active and absolute portfolios and is compared to reference results from the literature when using the nearest neighbour strategy (*G*_*NN*_) by [4, 10] and the universal portfolio strategy (UP) by [2].

**Table 8:** Wealth achieved by investing in combinations of stocks from NYSE dataset.

Stocks	Strat.	Wealth	Best Expert	Stocks	Strat.	Wealth	Best Expert
**IROQU** **KINAR**	Abs.	1.02e+12	1.76e+13	**COMME** **MEICO**	Abs.	3.56e+03	2.61e+04
	Act.	1.00e+11	4.97e+11		Act.	4.28e+01	2.49e+02
	*G*_*NN*_	1.16e+12	1.44e+13		*G*_*NN*_	3.51e+3	3.15e+4
	$G_{NN}^{*}$	1.01e+12	1.63e+13		$G_{NN}^{*}$	3.58e+03	2.60e+04
	UP	38.67			UP	72.63	
	Best	8.92			Best	52.02	
**COMME** **KINAR**	Abs.	2.99e+12	3.46e+13	**IBM** **COKE**	Abs.	7.84e+01	2.74e+02
	Act.	1.05e+11	3.75e+11		Act.	9.70e+00	1.98e+01
	*G*_*NN*_	4.78e+12	8.26e+13		*G*_*NN*_	74.37	296.3
	$G_{NN}^{*}$	3.09e+12	3.75e+13		$G_{NN}^{*}$	7.83e+01	2.67e+02
	UP	78.47			UP	14.18	
	Best	52.02			Best	13.36	
**36** **STOCKS**	Abs.	5.42e+01	1.36e+02				
	Act.	5.29e+01	7.13e+01				
	*G*_*NN*_	3.3e+11	7.7e+12				
	$G_{NN}^{*}$	3.43e+11	7.45e+12				
	Best	54.14					

Comparison of the total wealth achieved from the active (Act.) and absolute (Abs.) portfolios to the wealth achieved from the Györfi *et al* nearest neighbour (*G*_*NN*_), attempted recovery of the Györfi *et al* nearest neighbour ($G_{NN}^{*}$), the universal portfolio (UP) and a buy-and-hold of the best stock strategies.

$G_{NN}^{*}$ denotes our best recovery of the results of the nearest neighbour strategy [4, 10]. The results achieved by from the universal portfolio strategy was identically recovered [2]. The last row of Table 8 shows the results of the strategies when running on all 36 NYSE stocks.

The algorithm compares well to the two stocks combinations used in [2] and in [4, 10]. A surprising result is how the wealth achieved by the portfolio when run over all 36 stocks compares to the results in [4, 10], this may be due to a loss of accuracy in the quadratic approximation step of the algorithm as the number of stocks increase.

### NYSE merged data

**Fig 5:**
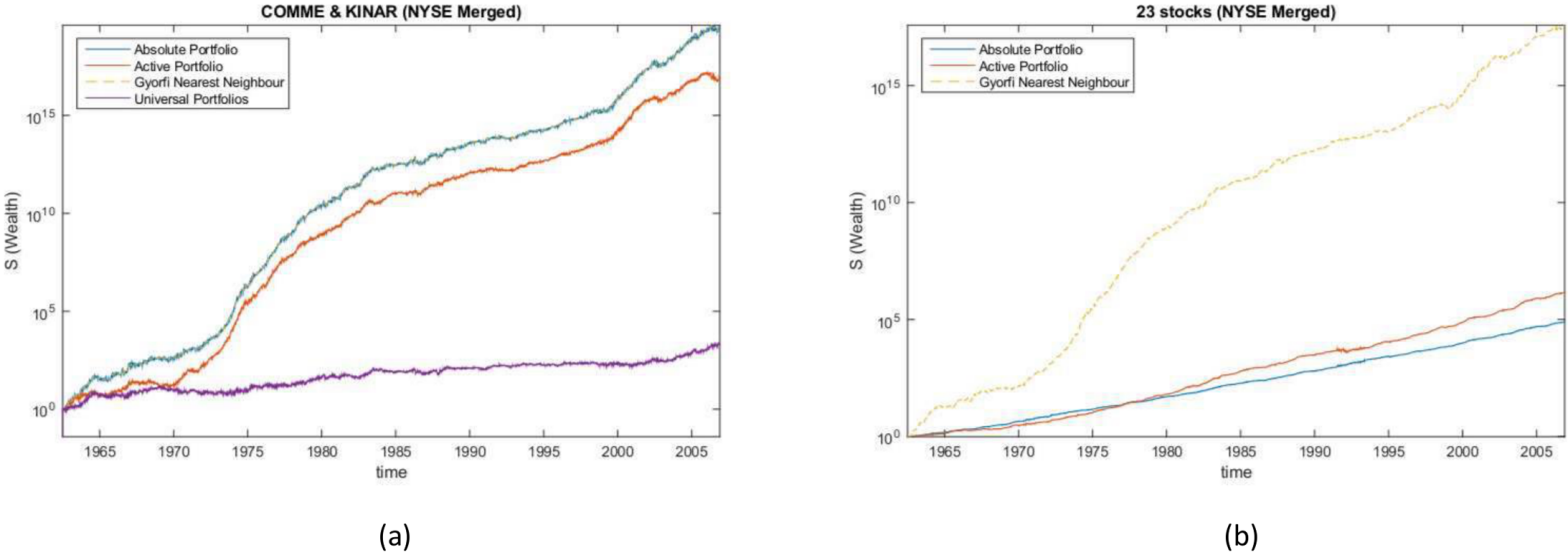
Wealth gained in NYSE merged data experiments. Comparison of the wealth gained from different methods when investing in (a) comme and kinar (b) 23 stocks from the NYSE Merged dataset.

The algorithm was run for both absolute and active portfolios on the NYSE Merged dataset on two stock combinations and on all of the 23 stocks in the dataset. The two stock combinations chosen were stocks Commercial Metals and Kin Ark Corp. and stocks IBM and Coca-Cola. Table 9 and Fig 5 shows the wealth achieved by the active and absolute portfolios compared to strategies from the literature.

**Table 9:** Wealth achieved by investing in various combinations of stocks from NYSE merged dataset.

Stocks	Strat.	Wealth	Best Expert
**COMME** **KINAR**	Abs.	3.07e+19	4.37e+20
	Act.	7.36e+16	7.93e+16
	$G_{NN}^{*}$	3.19e+19	4.73e+20
	UP	2192.43	
	Best	1344.3	
**IBM** **COKE**	Abs.	1.79e+03	5.12e+03
	Act.	7.79e+00	2.63e+01
	$G_{NN}^{*}$	1.79e+03	4.75e+03
	UP	229.13	
	Best	365.92	
**23** **STOCKS**	Abs.	8.05e+04	3.34e+05
	Act.	1.45e+06	5.42e+05
	$G_{NN}^{*}$	3.68e+17	5.60e+18
	Best	3496.7	

Comparison of the total wealth achieved from the active (Act.) and absolute (Abs.) portfolios to the wealth achieved from the attempted recovery of the Györfi *et al* nearest neighbour strategy ($G_{NN}^{*}$), the attempted recovery of the universal portfolio strategy (UP*) and a buy-and-hold strategy of the best stock (Best).

### Daily sampled JSE data

The algorithm was run for both absolute and active portfolios on various sets of two stock combinations, namely stocks AngloGold Ashanti Ltd and Anglo American PLC, stocks Standard Bank Group Ltd and FirstRand Ltd, and stocks Tiger Brands Ltd and Woolworths Holdings Ltd. The algorithm was also run for both absolute and active portfolios on a combination of 10 stocks, 20 stocks and 30 stocks (See S5 Appendix). In each case the date for which the data of a stock starts may be different, the time period for the algorithm therefore starts with the stock that has a later starting time. The JSE OHLC dataset was processed into four datasets containing *close-to-close*, *open-to-close*, *close-to-open* and *open-to-open* price relatives, the algorithm was run on each of these datasets.

**Fig 6:**
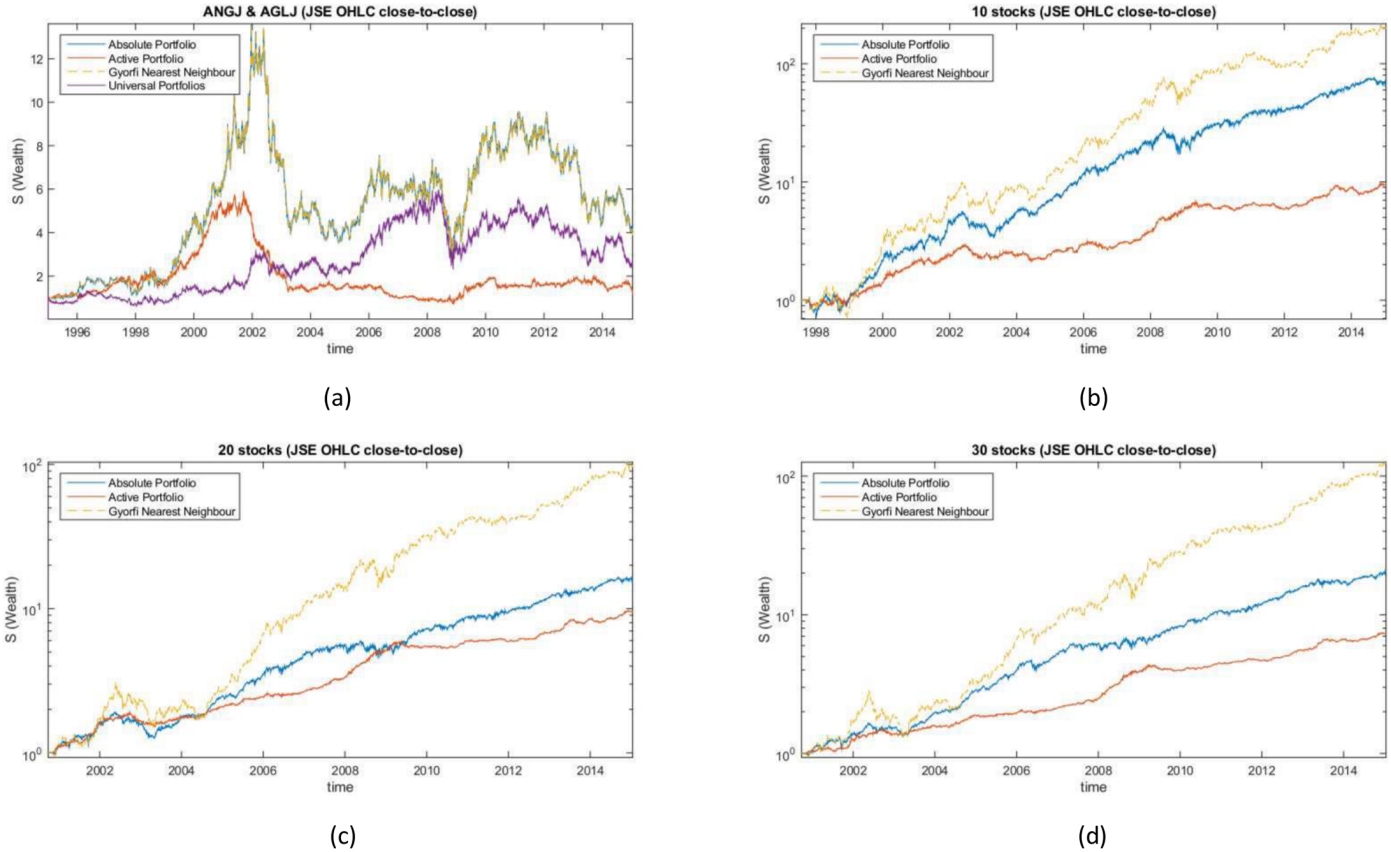
Wealth gained close-to-close JSE OHLC data. Comparison of the wealth gained from different methods when investing in (a) ANGJ and AGLJ (b) 10 stocks (c) 20 stocks (d) 30 stocks from the JSE OHLC *close-close* dataset. This does not account for price-impacts and frictions, nor for the need to approximate an expected close price just prior to market close as one solves for the portfolio controls, there will always be a difference between the controls solved for just prior to market close and those required once the market has closed and the official closing prices printed.

**Fig 7:**
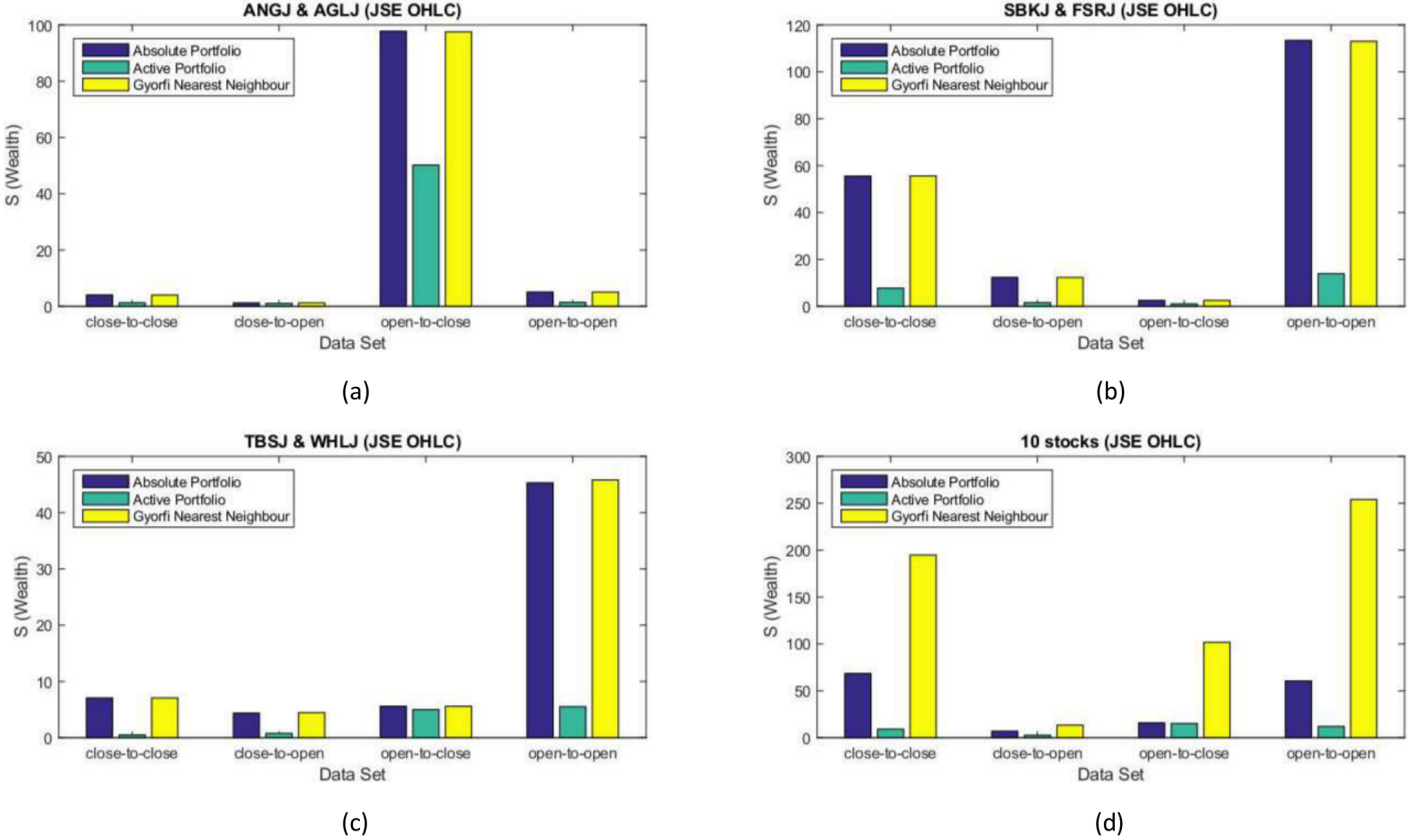
Wealth from Different JSE OHLC data sets. Comparison of wealth achieved from the absolute portfolio, active portfolio and Györfi nearest neighbour porfolio on the(a)*close-to-close* (b) *open-to-close* (c) *close-to-open* and (d) *open-to-open* JSE OHLC datasets. Here we find that there is no particular combination of OHLC data for which there is a systematic preference, e.g. *close-to-close*, the case of considering the close price change from one day end to another is not systematically more profitable than other combinations of data times. These tests do consider the reality of trading prior to a time point, for example market close, one cannot *a-priori* know what the close price will be, this has to be approximated. This excludes price-impact effects.

Tables 10, 11, 12 and 13 as well as Fig 6 shows the wealth achieved by the active and absolute portfolios compared to strategies from the literature. Fig 7 shows a comparison of the wealth achieved by the absolute, active and Györfi *et al* nearest neighbour strategy for the different price relative datasets (*close-to-close*, *open-to-close*, *close-to-open* and *open-to-open*).

**Table 10:** Wealth achieved by investing in various combinations of stocks from JSE OHLC dataset (close-to-close).

Stocks	Strat.	Wealth	Best Expert	Stocks	Strat.	Wealth	Best Expert
**ANGJ** **AGLJ**	Abs.	4.05	13.10	**SBKJ** **FSRJ**	Abs.	55.53	187.62
	Act.	1.28	3.40		Act.	7.77	13.40
	$G_{NN}^{*}$	4.02	13.27		$G_{NN}^{*}$	55.60	189.08
	UP	2.52			UP	18.17	
	Best	3.61			Best	21.09	
**TBSJ** **WHLJ**	Abs.	7.07	19.97	**10** **STOCKS**	Abs.	68.49	135.03
	Act.	0.49	1.80		Act.	9.28	12.84
	$G_{NN}^{*}$	7.07	19.49		$G_{NN}^{*}$	194.76	854.62
	UP	8.24					
	Best	8.97			Best	89.72	
**20** **STOCKS**	Abs.	16.20	63.18	**30** **STOCKS**	Abs.	20.48	58.27
	Act.	9.52	12.11		Act.	7.34	6.24
	$G_{NN}^{*}$	98.84	330.37		$G_{NN}^{*}$	124.91	590.51
	Best	89.72			Best	85.03	

The total wealth achieved by the active (Act.) and absolute (Abs.) portfolios compared to the wealth achieved from the attempted recovery of the Györfi *et al* nearest neighbour strategy ($G_{NN}^{*}$), the attempted recovery of the universal portfolio strategy (UP*) and a buy-and-hold strategy of the best stock (Best) on the *close-to-close* dataset.

**Table 11:** Wealth achieved by investing in various combinations of stocks from JSE OHLC dataset (close-to-open).

Stocks	Strat.	Wealth	Best Expert	Stocks	Strat.	Wealth	Best Expert
**ANGJ** **AGLJ**	Abs.	1.24	2.60	**SBKJ** **FSRJ**	Abs.	12.35	25.13
	Act.	1.04	2.82		Act.	1.57	3.06
	$G_{NN}^{*}$	1.24	2.56		$G_{NN}^{*}$	12.31	25.14
	UP	0.97			UP	7.89	
	Best	2.66			Best	12.47	
**TBSJ** **WHLJ**	Abs.	4.38	9.00	**10** **STOCKS**	Abs.	7.11	11.43
	Act.	0.79	1.88		Act.	2.68	3.39
	$G_{NN}^{*}$	4.46	8.83		$G_{NN}^{*}$	13.45	30.53
	UP	4.89					
	Best	4.98			Best	56.36	
**20** **STOCKS**	Abs.	8.82	16.87	**30** **STOCKS**	Abs.	8.07	15.80
	Act.	5.28	5.24		Act.	5.66	5.50
	$G_{NN}^{*}$	11.00	27.01		$G_{NN}^{*}$	22.53	54.80
	Best	56.36			Best	51.02	

The total wealth achieved by the active (Act.) and absolute (Abs.) portfolios compared to the wealth achieved from the attempted recovery of the Györfi *et al* nearest neighbour strategy ($G_{NN}^{*}$), the attempted recovery of the universal portfolio strategy (UP*) and a buy-and-hold strategy of the best stock (Best) on the *close-to-open* dataset.

**Table 12:** Wealth achieved by investing in various combinations of stocks from JSE OHLC dataset (open-to-close).

Stocks	Strat.	Wealth	Best Expert	Stocks	Strat.	Wealth	Best Expert
**ANGJ** **AGLJ**	Abs.	97.75	395.58	**SBKJ** **FSRJ**	Abs.	2.53	5.12
	Act.	50.21	91.82		Act.	1.04	1.80
	$G_{NN}^{*}$	97.53	398.74		$G_{NN}^{*}$	2.53	5.00
	UP	3.41			UP	2.66	
	Best	4.68			Best	2.97	
**TBSJ** **WHLJ**	Abs.	5.59	12.73	**10** **STOCKS**	Abs.	15.90	33.40
	Act.	5.00	7.19		Act.	15.21	19.86
	$G_{NN}^{*}$	5.59	12.75		$G_{NN}^{*}$	101.80	415.97
	UP	1.94					
	Best	1.89			Best	169.83	
**20** **STOCKS**	Abs.	4.80	8.88	**30** **STOCKS**	Abs.	5.16	11.98
	Act.	6.80	6.07		Act.	4.58	5.57
	$G_{NN}^{*}$	30.26	104.46		$G_{NN}^{*}$	50.17	317.92
	Best	169.83			Best	1278.83	

The total wealth achieved by the active (Act.) and absolute (Abs.) portfolios compared to the wealth achieved from the attempted recovery of the Györfi *et al* nearest neighbour strategy ($G_{NN}^{*}$), the attempted recovery of the universal portfolio strategy (UP*) and a buy-and-hold strategy of the best stock (Best) on the *open-to-close* dataset.

**Table 13:** Wealth achieved by investing in various combinations of stocks from JSE OHLC dataset (open-to-open).

Stocks	Strat.	Wealth	Best Expert	Stocks	Strat.	Wealth	Best Expert
**ANGJ** **AGLJ**	Abs.	5.11	26.65	**SBKJ** **FSRJ**	Abs.	113.43	412.75
	Act.	1.43	9.10		Act.	13.97	18.87
	$G_{NN}^{*}$	5.07	25.66		$G_{NN}^{*}$	113.02	415.45
	UP	2.28			UP	20.29	
	Best	3.18			Best	20.55	
**TBSJ** **WHLJ**	Abs.	45.30	266.17	**10** **STOCKS**	Abs.	60.62	342.40
	Act.	5.52	28.54		Act.	12.03	20.57
	$G_{NN}^{*}$	45.80	273.44		$G_{NN}^{*}$	254.00	2292.47
	UP	8.81					
	Best	9.24			Best	69.49	
**20** **STOCKS**	Abs.	14.73	73.61	**30** **STOCKS**	Abs.	21.54	73.88
	Act.	9.13	14.76		Act.	11.48	18.89
	$G_{NN}^{*}$	44.88	192.99		$G_{NN}^{*}$	161.16	1069.81
	Best	69.49			Best	131.89	

The total wealth achieved by the active (Act.) and absolute (Abs.) portfolios compared to the wealth achieved from the attempted recovery of the Györfi *et al* nearest neighbour strategy ($G_{NN}^{*}$), the attempted recovery of the universal portfolio strategy (UP*) and a buy-and-hold strategy of the best stock (Best) on the *open-to-open* dataset.

### Intraday JSE data

**Fig 8:**
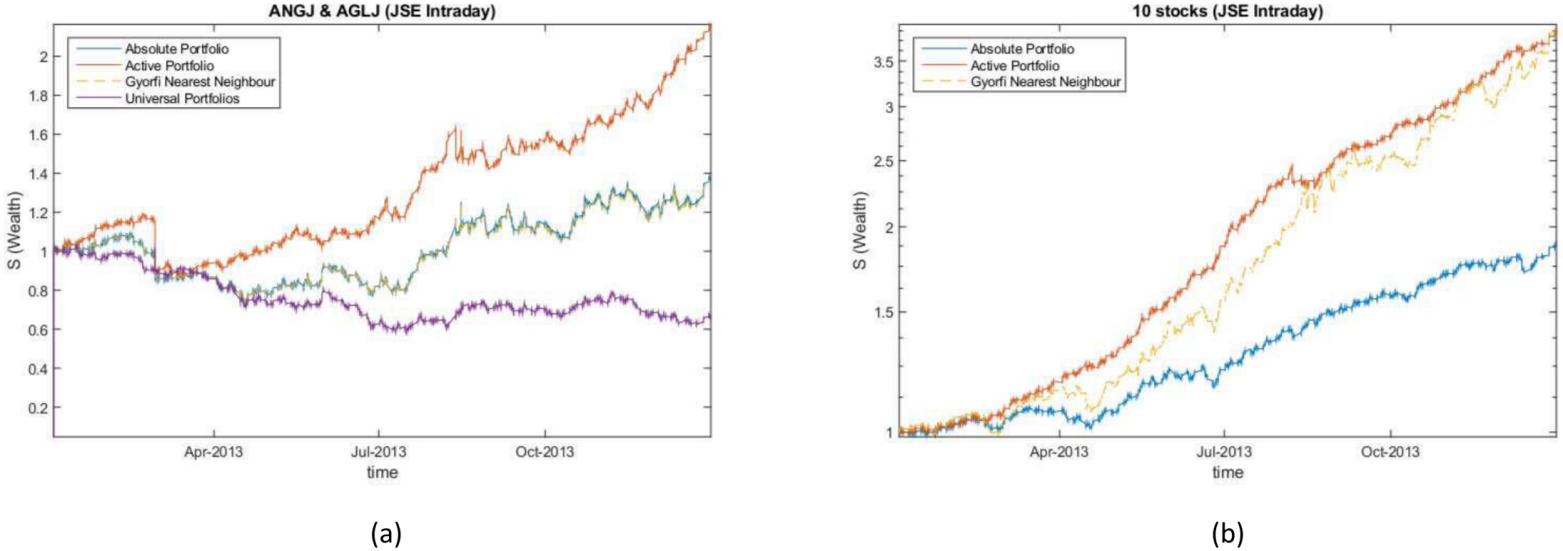
Wealth from JSE intraday data experiments. Comparison of the wealth gained from different methods when investing in (a) ANGJ and AGLJ (b) 10 stocks from the JSE Intraday dataset.

The algorithm was run for both absolute and active portfolios on various sets of two stock combinations, namely stocks AngloGold Ashanti Ltd and Anglo American PLC, stocks Standard Bank Group Ltd and FirstRand Ltd, stocks Tiger Brands Ltd and Woolworths Holdings Ltd, and stocks MTN Group Ltd and Vodacom Group Ltd. The algorithm was also run for both absolute and active portfolios on the a combination of of 10 stocks, 20 stocks and 30 stocks (See S5 Appendix). Table 14 and Fig 8 shows the wealth achieved by the active and absolute portfolios compared to strategies from the literature.

**Table 14:** Wealth from stock-pairs of JSE intraday data.

Stocks	Strat.	Wealth	Best Expert	Stocks	Strat.	Wealth	Best Expert
**ANGJ** **AGLJ**	Abs.	1.38	3.33	**SBKJ** **FSRJ**	Abs.	1.82	2.19
	Act.	2.16	5.01		Act.	2.02	2.01
	$G_{NN}^{*}$	1.36	3.21		$G_{NN}^{*}$	1.82	2.15
	UP	0.66			UP	1.08	
	Best	0.87			Best	1.08	
**TBSJ** **WHLJ**	Abs.	1.95	3.06	**MTNJ** **VODJ**	Abs.	2.13	3.55
	Act.	2.24	3.29		Act.	2.13	3.25
	$G_{NN}^{*}$	1.95	2.99		$G_{NN}^{*}$	2.13	3.57
	UP	0.91			UP	1.12	
	Best	0.99			Best	1.18	
**10** **STOCKS**	Abs.	1.89	2.79	**20** **STOCKS**	Abs.	1.74	2.35
	Act.	3.86	5.40		Act.	3.42	4.29
	$G_{NN}^{*}$	3.95	14.05		$G_{NN}^{*}$	5.68	21.62
	Best	1.93			Best	1.93	
**30** **STOCKS**	Abs.	1.67	2.16				
	Act.	3.07	3.76				
	$G_{NN}^{*}$	6.03	12.61				
	Best	1.93					

The total wealth achieved by the active (Act.) and absolute (Abs.) portfolios compared to the wealth achieved from the attempted recovery of the Györfi *et al* nearest neighbour strategy ($G_{NN}^{*}$), the attempted recovery of the universal portfolio strategy (UP*) and a buy-and-hold strategy of the best stock (Best) on the JSE Intraday Dataset.

**Fig 9:**
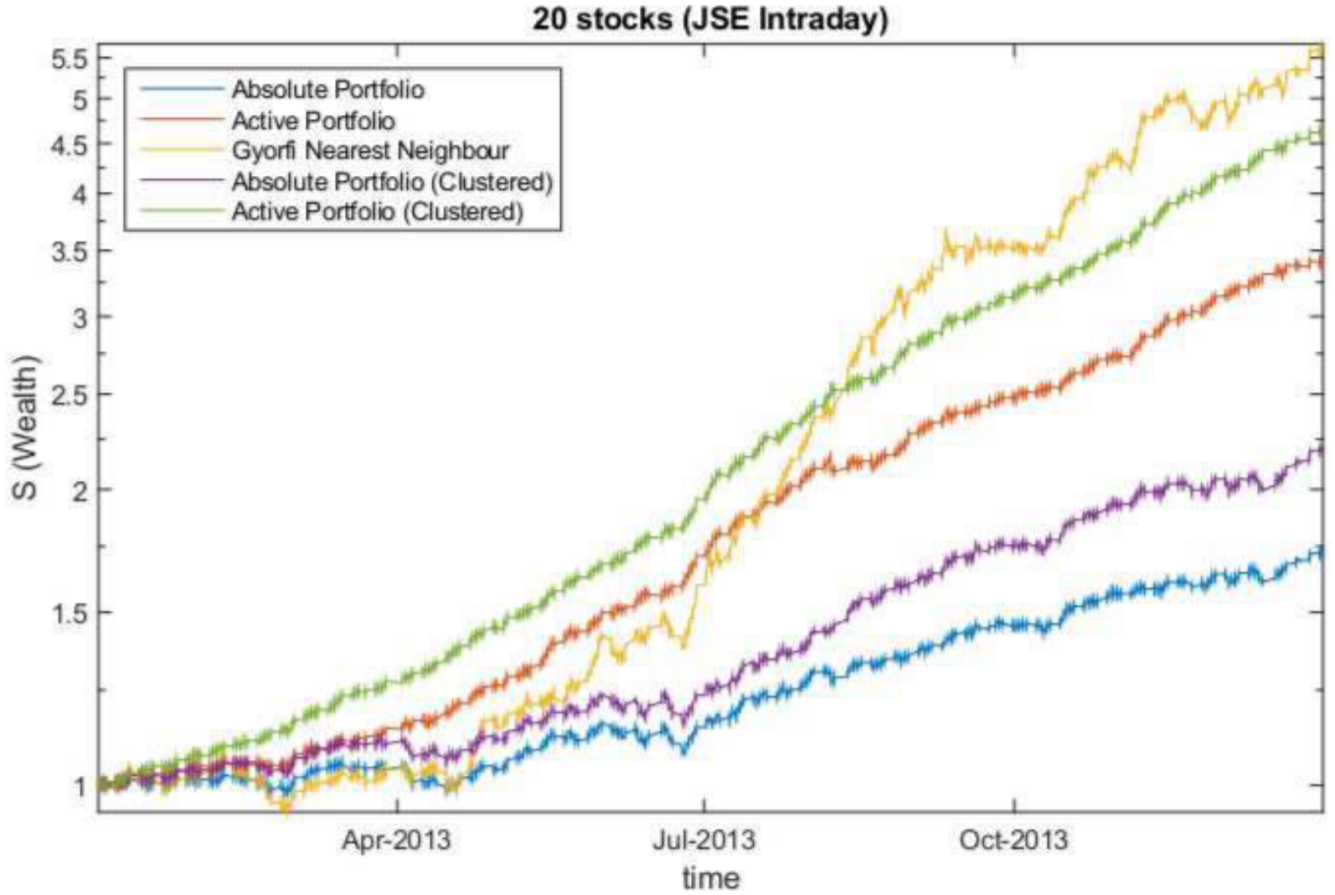
20 JSE stocks. Comparison of the wealth gained from different methods when investing in 20 stocks from the JSE Intraday dataset, the plot includes the results of using clusters on the stocks. It is important to note that the clustered portfolios have 150 experts and the portfolios without clusters have 50 experts.

Table 15 and Fig 9 shows the wealth achieved by active and absolute portfolios when using economic sectors as clusters.

**Table 15:** JSE intraday data with cluster defined experts.

		RESI	INDI	FINI
Act.	Best Expert	9.1018	3.4470	3.6698
	Total Wealth	4.6368		
Abs.	Best Expert	5.6655	2.6341	2.6059
	Total Wealth	2.2093		

Wealth achieved by active and absolute portfolios when using economic sectors as clusters. Using three clusters increases the number of competing experts by a factor of 3, from 50 to 150. The inclusion of a larger set of experts increase the out-of-sample wealth performance of the two strategies. See S5 Appendix for the particular stocks in each sectors, Resources (RESI), Financials (FINI) and Industrials (INDI), respectively. The total wealth includes the relative competition between experts defined by the three economic sectors. The sector specific wealth is given the best performing experts from each cluster groups of stocks.

### Impact of market frictions

An important criticism of any strategy simulation relates to the need to account for the impact of market frictions both direct and indirect [22], these can include: transaction costs, the cost of the capital for trading, the cost of market access, the cost of regulatory capital for taking risky trading positions, and market impact. These are all required to be included in any estimate of performance for any realistic assessment of the viability of trading activity. Here we have focussed our attention on transaction costs and estimates of permanent market impact. Once one considers the impact of settlement, administrative, technology and compliance costs things become more complex. We have focussed on transaction costs alone as the implementation is considered in the context of a business use case that is associated with bulk trading activities such as those associated with a market maker, hedge-fund or various institutional trading activities.

#### Daily strategy trading frictions

The argument that the zero-cost low frequency (daily traded) strategies are viable, even when unleveraged, is based on the 7 years of history. Although this may be considered short in the context of many academic studies, this is of the order of the time-scale of the business cycle so we considered this realistic. Consider Table 10 for the active case for the Top 10 JSE stocks (see S5 Appendix). Here we would argue for 15bps of daily profit before costs (from Table 10 using the accumulated daily wealth of 9.53). We consider the strategy that trades close of the one day to the close of the next day (close-to-close).

This is considered in order to take into account liquidity effects. The closing auction is the most liquid time to trade on the JSE. It is unlikely that one would be able to achieve low slippage trading near the daily market opening. We consider the combination of cost of capital (the borrowing costs required to source trading capital and cost of regulatory capital) and a small penalty for slippage due to the differences between the realized closing prices and the estimated closing prices that the algorithm would require in order to estimate the portfolio controls as 10bps per day. This can in practice be carried out during the closing auction, just prior to the market close, by estimating online, the equilibrium price that could be the result of maximizing execution volume for the lowest surplus when the market is cleared at market close, bearing in mind that there is a short randomization period at the end of the auction that needs to be accounted for. It is fairly straight-forward to estimate sufficiently reasonable market clearing prices. If this is not considered realistic, one can then merely consider the trading to have occurred during a post market close period (such as that found on the LSE and JSE) where one can transact at the market close price—but at lower volumes. In practise it should also be noted that such a trading strategy can be converted to one that trades in equity swaps, so-called contracts-for-difference (CFDs), this would convert the uncertainty about slippage and costs into an up-front fee and allow for good implementation of the required model positions with a known direct cost and no meaningful liquidity concerns. If the daily strategy was implemented with these types of delta-one instruments our estimates of costs can then be considered conservative.

We argue that we can realistically earn a modest 5bps of unleveraged self-funded trading profit per day, or an annual return of 12% of unleveraged profit-and-loss; but only if we consider direct transaction costs [32].

#### Intraday strategy trading costs

The direct intraday costs can be estimated from the strategy turn-over. Our indicative direct cost of trading assumed that we have 100% turn-over of inventory at each trading period with a consistent cost of 0.55bps (0.0055%) [32] per trading period with an additional 4bps to give an indicative slippage of 50bps for the intraday trading per day. Direct costs can be expected to be at least 50bps per day. This excludes the cost of borrowing capital to trade. If we considered trading to be for a 7 hour period starting a half hour after market opening, and stopping 15 minutes before the market closing auction (using the JSE market times), this then leads to 84 = 12 × 7 5-minute trading periods across the day, this would give the worst case scenario of 8400% turn-over per day, at a direct cost of 0.55bps per trading period, we argue that this then leads to direct costs of 46bps per day, additional frictions of 4bps are added to this to get the over-all daily direct cost estimate of 50bps per day.

For our heuristic argument we assume: 1.) a daily direct cost (fees) of 50bps for the (self-financing) zero-cost statistical arbitrage strategy, 2.) borrowing costs on the capital required for trading over the year to be 10%, and 3.) that the strategy we denote as the active strategy generated a 4.63 wealth gained over a year of trading (see Table 15). Putting these together we argue for an upper limit on the profit, even when unleveraged, to be a return of 20% for 250 days of trading. The strategy generates 62bps per day, the direct costs of 50bps, leaving 12bps, this less the 5bps for the cost of capital leaves 7bps to accumulate as profit-and-loss per trading day. If we then include a spread greater than 7bps (perhaps using aggressive implementation) we are losing money.

The indirect intraday costs that could arise from market impact can be estimated using the square-root law as a “rule-of-thumb”: $cost=spread+\sigma \sqrt{n/ADV}$ [33]. Here we argue for: (i.) a conservative and, (ii.) a moderate scenario. For the conservative scenario we argue that we can restrict our trading to no more than 5% of the average daily volume traded (ADV) and that the average daily spread is no more than 12bps, with a daily volatility of 3% (for sake of argument) to find a cost of 80bps; while the moderate scenario argues for a spread of 9bps and a daily volatility of near to 2%, where one finds a cost of 50bps. Indirect costs can be expected to be between 50bps and 80bps per day.

We conclude that one needs to realistically expect intraday transaction costs to be at least somewhere between 50bps and 100bps per day. We draw the same conclusions, that even with optimistic costs for the strategy return being close to 65bps, this is on the boundary of being profitable even before indirect costs are included.

It may well be that the kind of pattern driven strategies that the pattern matching algorithm exploits is driven by artefacts in the financial time-series data that cannot generally be profitably arbitraged away. Irrespective of the reason for the existence of persistent patterns, we have demonstrated that such patterns do exist; but we do not provide evidence that the patterns can be profitably exploited.

#### Business model costs

We have based much of the above argument on transaction costs on the JSE; direct costs using the 0.0055% cost from the combined minimum of transaction cost per trading leg [32] and indirect costs based on the square-root law. However, in the context of the JSE the real cost of trading can be incurred at the business model level if the business is not carefully included with bulk trading activities.

The costs associated with the Securities Transfer Tax (STT) [32] and various taxes and administrative costs are the most onerous. For example, one can expect to pay the following costs: 1. business related administration fee (which we ignore), 2. brokerage fee (we ignore because we argue that with bulk institutional trading this can be ignored), 3. JSE transaction costs per trade: 0.0053% (0.53bps) per trade, 4. JSE Investor Protection Levy (IPL): 0.0002% (2bps) per trade, 5. settlement fee JSE STRATE: 0.0036% (one may be able implement this at the end of a trading day through bulk trading), 4. Securities Transfer Tax (STT): 0.25%, 7. JSE Value-Added-Tax (VAT): 14% (brokerage, IPL and STRATE) (paid to the South African Revenue Services (SARS)), 8. Capital Gains Tax (CGT), also paid to the tax revenue services and 9. a custodian fee (which we ignore here). We only consider costs 3 and 4 with some additional frictions to account from the spread and the cost of capital. So realistically one cannot avoid at least 50bps to 80bps per day before accounting for STT [32].

We have also excluded settlement costs (which can be estimated from the end of day positions if trading is well structured). However, on the JSE STT is 0.25%, this alone will ensure that the strategies considered here in the context of the JSE are probably unviable without being part of bulk trading activities. If this is indeed the case, it provides a possible explaination for why these types of statistical arbitrages can be so persistent and remain in the JSE time-series data investigated.

## Conclusion

In prior work it has been shown that in South African financial markets persistence and long-memory are generic [34]. This paper adds to our knowledge of the South African market by showing that in addition to evidence supporting long-memory processes, price processes have patterns that are predictable in a straight-forward manner. We argue that predictability does not necessarily imply profitability; although this is not in itself new, the context in which we have replicated both the intraday and daily strategies is.

We present a simple established portfolio value based learning algorithm, a multi-manager in the language of asset management, that selects an over-all portfolio with weights ***b*** by considering a selection of N different strategies ***H***_*n*_. The expert strategy portfolio weights are constructed for underlying strategies that are enumerated over a variety of combinations of time-series patterns, time-scales, clusters and partitions. This is considered in the context of universally consistent strategies [2, 4] but with an extension to directly consider self-financing zero-cost quantitative trading strategies. When applying the algorithms to real daily test data they compare well to results from our replication of algorithms from the literature [2, 4] and actual results from the literature from the New York Stock Exchange (NYSE) dataset (see Tables 8 and 9).

The active zero-cost version of the algorithm when applied to intraday data from the Johannesburg Stock Exchange (JSE) is shown to have performed well in comparison to the best stock, and compares favourably with methods from prior work [2, 4] (see Table 14). We show that on the Johannesburg Stock Exchange data the algorithms can learn trends and patterns and enhance out-of-sample wealth accumulation for both daily and intraday applications (see Tables 10 and 14). This is demonstrated on both low frequency data, daily sampled data, and higher frequency data, intraday uniformly sampled transaction data.

We have shown that there is an advantage to include experts that are clustered on stock economic sector classifications (see Table 15); this increases the number of experts considered by the learning algorithm through including sector membership into the resource, financial and industrial stocks sectors, which in turn boosts the out-of-sample performance. This suggests that combining more sophisticated clustering algorithms [27] with machine learning can be advantageous in the domain of quantitative trading.

**Fig 10:**
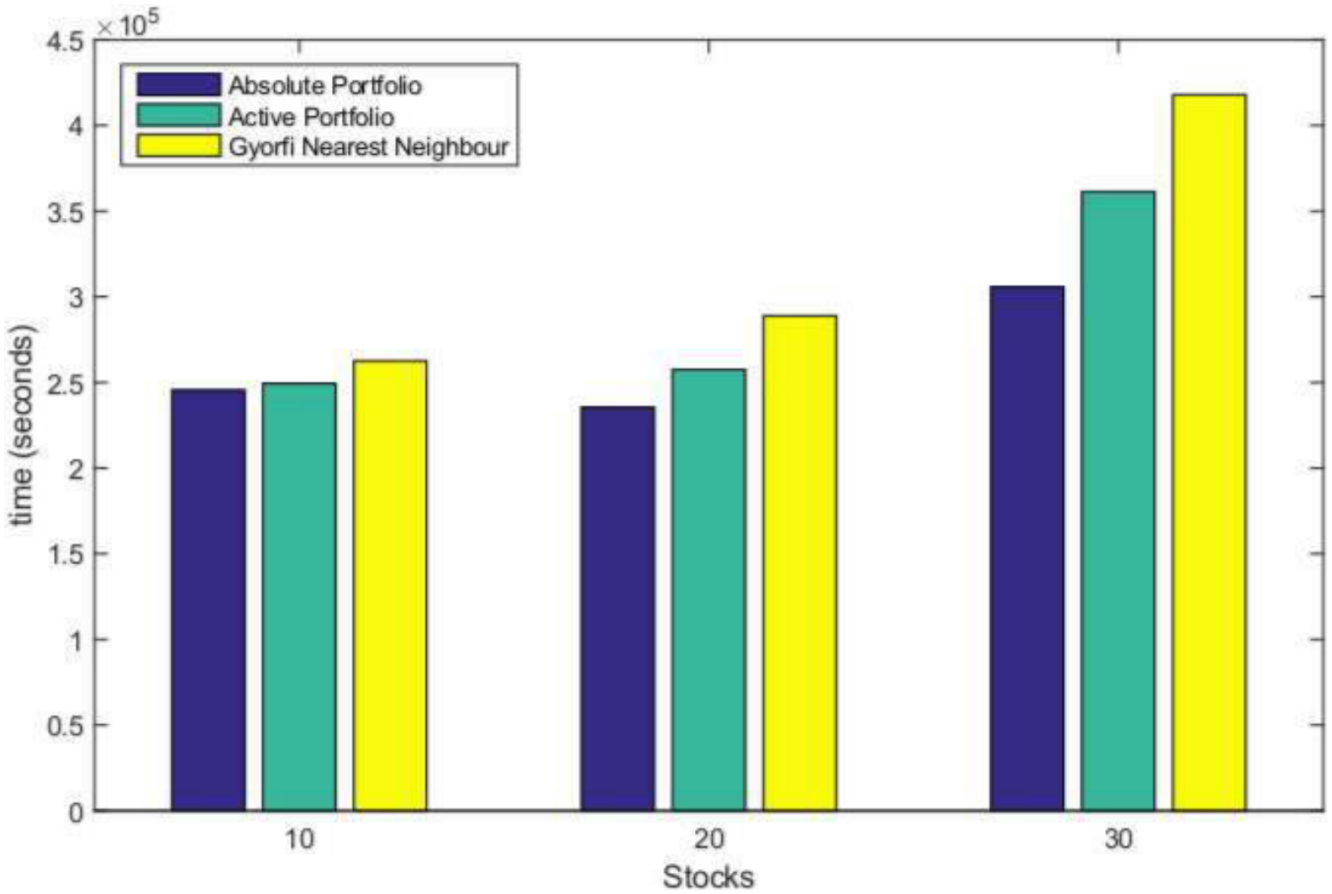
JSE intraday data running times. Running time of the portfolios in seconds of the different strategies. This demonstrates the speed advantage of using the analytic quadratic approximation as compared to numerically solving the log-optimal constrained optimization at each time-step for each expert combination. As expected the fully invested analytic solution is fastest, the zero-cost portfolio next, because of the additional leverage constraint, and the slowest the algorithm that required the numerical solution of the optimization.

The pay-off between computational performance and wealth accumulation can be seen by considering the increased duration of the simulation as one increases from 10 stocks, to 20 stocks through to 30 stocks in Fig 10. The commensurate loss in performance can be seen in Table 14. For example, the 20 stock simulation generated a wealth of 1.74 for the absolute portfolio and 5.68 for the Gyorfi *et al* nearest-neighbour strategy with the absolute portfolio being almost 5 × 10^4^ seconds faster (or 18% faster). For intraday statistical arbitrage problems for quantitative trading with many (50>) assets, computational delays can lead to lags between information arrival and order-execution that can negatively impact a strategies profit-and-loss performance.

We have shown that in the daily dataset for the Johannesburg Stock Exchange, when considering open, high, low and close price data, there is an advantage when considering strategies that relate to the patterns arising across closing price to closing price data (see Table 10). This provides evidence that one can in principle beat the best stock (or the money market account in the case of the self-financing strategy) as pattern persistence is sufficiently robust in the markets considered. However, it is difficult to profitable trade the market opening price to the market closing price as intraday dynamics seems to become important and one tends to incur significant trading costs associated with poor market liquidity near the market opening.

The impact of both direct and indirect transaction costs are onerous (see Impact of Market Frictions section). Our estimate for the impact of transactions costs on the intraday strategy leave an optimistic annual return of upto 20% for the unleveraged self-financing strategy if we exclude indirect costs—if we include indirect costs we are probably losing money. Using daily sampled strategy to trade the closing price of the market from one day to the next can generate an optimisitc annual return of upto 12% if indirect costs can be effectively managed. Using this we argue that the self-financing zero-cost portfolio strategy can be considered tractable across days from the perspective of transaction costs. However, as a business model, estimating the correct costs is more complex as one needs to include additional costs such as settlement costs, value-added tax, capital gains tax, securities transfer taxes and technology costs in the context of the market considered [32] as well as the cost of raising trading capital.

It should be noted that for optimised intraday risk trading the event-time version of the strategy should be implemented rather than the calendar-time approach. The calendar-time approach was used for simplicity in the experiments in this paper. This is fairly straight-forward to implement using equal volume buckets and the online down-sampling of transaction data to a time-series of volume-weighted average prices for equal volume buckets [35]. However, due to the realities of cost layering it still remains unlikely that these types of strategies can be profitable without well structure market access. For market participants with incentives for market-making the situation may be very different if these types of intraday strategies are combined with market-making models.

Their are a variety of barriers to entry relating to both reasonable and cost effect market access, as well as the scalability of these types of strategies due to stock liquidity. In terms of market access many proprietary trading structures within hedge-funds and banks would have low combinations of transaction, settlement and technology costs due to bulk trading activities. For this reason we consider our daily transaction costs of 80bps as optimistic. In terms of the liquidity concerns, we have limited ourselves, in the Johannesburg Stock Exchange data set, to collections of the 10 and 20 most liquid stocks. These stocks can be traded in volumes useful to typical business models for proprietory trading. This support the central thesis of the research the patterns we identify may be predictable but this does not imply profitabliity (even after transaction costs have been accounted for) without careful considerations for the business model structure for trading. We argue that our work supports the general argument that fairly naive data-informed computational learning experts with the appropriate access to the systems can generate wealth (or at least cover the cost of trading) without special insights.

## Supporting information

S1 Appendix The learning algorithm. This is discussed in the An Online-Learning Algorithm for Portfolio Selection section.
(TEX)

S2 Appendix The pattern selection algorithm. This is discussed in the Expert Generating Algorithms from Patterns section and the Pattern-Matching section and uses the matching algorithm described below in S3 Appendix. This algorithm iteratively presents segments of data to the find best matches with past data.
(TEX)

S3 Appendix The matching algorithm. This is discussed in the Expert Generating Algorithms from Patterns section and the Pattern-Matching section and is used in the pattern-matching algorithm described above in S2 Appendix. This algorithm finds the best match for a particular segment of data.
(TEX)

S4 Appendix NYSE ticker list. (TEX)

S5 Appendix JSE intraday and daily data ticker list. In the following tables ✓* represents stocks that are included in the JSE daily dataset grouping and not the JSE intraday dataset grouping, and ✓^+^ represents stocks that are include in the JSE intraday dataset grouping and not the JSE daily dataset grouping.
(TEX)
